# Reconstructing Asian faunal introductions to eastern Africa from multi-proxy biomolecular and archaeological datasets

**DOI:** 10.1371/journal.pone.0182565

**Published:** 2017-08-17

**Authors:** Mary E. Prendergast, Michael Buckley, Alison Crowther, Laurent Frantz, Heidi Eager, Ophélie Lebrasseur, Rainer Hutterer, Ardern Hulme-Beaman, Wim Van Neer, Katerina Douka, Margaret-Ashley Veall, Eriéndira M. Quintana Morales, Verena J. Schuenemann, Ella Reiter, Richard Allen, Evangelos A. Dimopoulos, Richard M. Helm, Ceri Shipton, Ogeto Mwebi, Christiane Denys, Mark Horton, Stephanie Wynne-Jones, Jeffrey Fleisher, Chantal Radimilahy, Henry Wright, Jeremy B. Searle, Johannes Krause, Greger Larson, Nicole L. Boivin

**Affiliations:** 1 Radcliffe Institute for Advanced Study, Harvard University, Cambridge, MA, United States of America; 2 Faculty of Life Sciences, University of Manchester, Manchester, United Kingdom; 3 School of Social Science, The University of Queensland, Brisbane Queensland, Australia; 4 Palaeogenomics & Bio-Archaeology Research Network, Oxford University, Oxford, United Kingdom; 5 School of Biological and Chemical Sciences, Queen Mary University of London, Mile End Road, London, United Kingdom; 6 Dept. Ecology and Evolutionary Biology, Cornell University, Ithaca, NY, United States of America; 7 Research Laboratory for Archaeology and the History of Art, Oxford University, Oxford, United Kingdom; 8 Dept. Vertebrates, Zoologisches Forschungsmuseum Alexander Koenig, Bonn, Germany; 9 Dept. Archaeology, University of Aberdeen, Aberdeen, United Kingdom; 10 Royal Belgian Institute of Natural Sciences, Brussels, Belgium; 11 Dept. Biology, University of Leuven, Leuven, Belgium; 12 Dept. Anthropology, Rice University, Houston, United States of America; 13 Institute for Archaeological Sciences, University of Tübingen, Tübingen, Germany; 14 Canterbury Archaeological Trust, Canterbury, United Kingdom; 15 McDonald Institute for Archaeological Research, Cambridge, United Kingdom; 16 British Institute in Eastern Africa, Nairobi, Kenya; 17 Dept. Zoology, Osteology Section, National Museums of Kenya, Nairobi, Kenya; 18 Dept. Systématique & Evolution, Muséum National d’Histoire Naturelle, Paris, France; 19 Dept. Archaeology and Anthropology, University of Bristol, Bristol, United Kingdom; 20 Dept. Archaeology, University of York, York, United Kingdom; 21 Musée d’Art et d’Archéologie, Université d’Antananarivo, Antananarivo, Madagascar; 22 Museum of Anthropology, University of Michigan, Ann Arbor, United States of America; 23 Santa Fe Institute, Santa Fe NM, United States of America; 24 Max Planck Institute for the Science of Human History, Jena, Germany; Seoul National University College of Medicine, REPUBLIC OF KOREA

## Abstract

Human-mediated biological exchange has had global social and ecological impacts. In sub-Saharan Africa, several domestic and commensal animals were introduced from Asia in the pre-modern period; however, the timing and nature of these introductions remain contentious. One model supports introduction to the eastern African coast after the mid-first millennium CE, while another posits introduction dating back to 3000 BCE. These distinct scenarios have implications for understanding the emergence of long-distance maritime connectivity, and the ecological and economic impacts of introduced species. Resolution of this longstanding debate requires new efforts, given the lack of well-dated fauna from high-precision excavations, and ambiguous osteomorphological identifications. We analysed faunal remains from 22 eastern African sites spanning a wide geographic and chronological range, and applied biomolecular techniques to confirm identifications of two Asian taxa: domestic chicken (*Gallus gallus*) and black rat (*Rattus rattus*). Our approach included ancient DNA (aDNA) analysis aided by BLAST-based bioinformatics, Zooarchaeology by Mass Spectrometry (ZooMS) collagen fingerprinting, and direct AMS (accelerator mass spectrometry) radiocarbon dating. Our results support a late, mid-first millennium CE introduction of these species. We discuss the implications of our findings for models of biological exchange, and emphasize the applicability of our approach to tropical areas with poor bone preservation.

## Introduction

Human trade, travel, and transport have facilitated the translocation of a vast number of species around the world, creating cosmopolitan assemblages of organisms across all continents [1]. Perhaps the best-known historical example is the Columbian Exchange, in which a broad range of domesticated plants, animals, weeds, and diseases crossed the Atlantic in both directions in the years after 1492, transforming human demography, natural landscapes, and economic systems [2]. Earlier exchanges elsewhere in the world have also been postulated [3], and here we consider the so-called ‘Monsoon Exchange’ that resulted in the movement of plant and animal species between Asia and Africa [4]. The details of this exchange remain poorly understood, due to a paucity of systematic archaeobotanical, zooarchaeological, and chronometric studies, particularly in Africa.

The Swahili coast–a cultural region stretching from southern Somalia to northern Mozambique and including near-shore islands, as well as the Comoros and Madagascar–is a key area for exploring human-mediated biological exchange between Asia and Africa. Historical and archaeological sources testify to the long-term engagement of the Swahili coast with the wider Indian Ocean world, through commercial and cultural interactions that promoted the emergence of a cosmopolitan, trade-oriented society by the late first millennium CE [5]. Biological exchange was part of the Swahili phenomenon, leading to the development, for example, of agricultural systems in which Asian domesticates play a key role [6–8].

Two broad models are proposed for Asian species introductions along the Swahili coast. One posits an arrival no earlier than the 6^th^ century CE, coincident with the first clear evidence for Indian Ocean trade at coastal sites in the form of imported ceramics, glass beads, and metal objects. This model links the arrival of species such as black rat (*Rattus rattus*), house mouse (*Mus musculus*), zebu cattle (*Bos indicus*), domestic chicken (*Gallus gallus*), banana (*Musa* spp.), and Asian rice (*Oryza sativa*) to historically-documented trade with the Middle East and Asia [9–11]. This model is undermined by Classical descriptions (in sources such as the first century CE *Periplus of the Erythraean Sea*) of an earlier well-established trade between Arabia and the eastern African coast [12]. Attempts to link these descriptions to archaeological findings on the Swahili coast (e.g., [13, 14]), however, have produced tenuous and controversial evidence [5] and contested identifications of translocated species [15].

A second model proposes a much earlier arrival in eastern Africa of one or more Asian species, drawing on archaeological and other lines of evidence. Dates as early as the late fourth millennium BCE have been claimed for domestic chicken on Zanzibar [16, 17], for example. An early arrival and westward spread of the banana has also been postulated [18], based on the recovery of phytoliths from sites in Uganda (late fourth millennium BCE [19]) and Cameroon (mid-first millennium BCE [20]). Food production is not conventionally thought to have spread to the eastern African coast from the interior until the early first millennium CE with the arrival of Bantu-speaking farmers. However supporters of the early model have argued for the plausibility of pre-Iron Age coastal populations capable of the cultivation and husbandry of domesticates introduced from Asia [18, 21]. This model of early Asian introductions has been challenged on the basis of taxonomic identifications inadequately supported by morphometric or other criteria, problematic dating and/or stratigraphic control, and a reliance on rare findings not replicated by subsequent studies [15, 22, 23].

**Fig 1:**
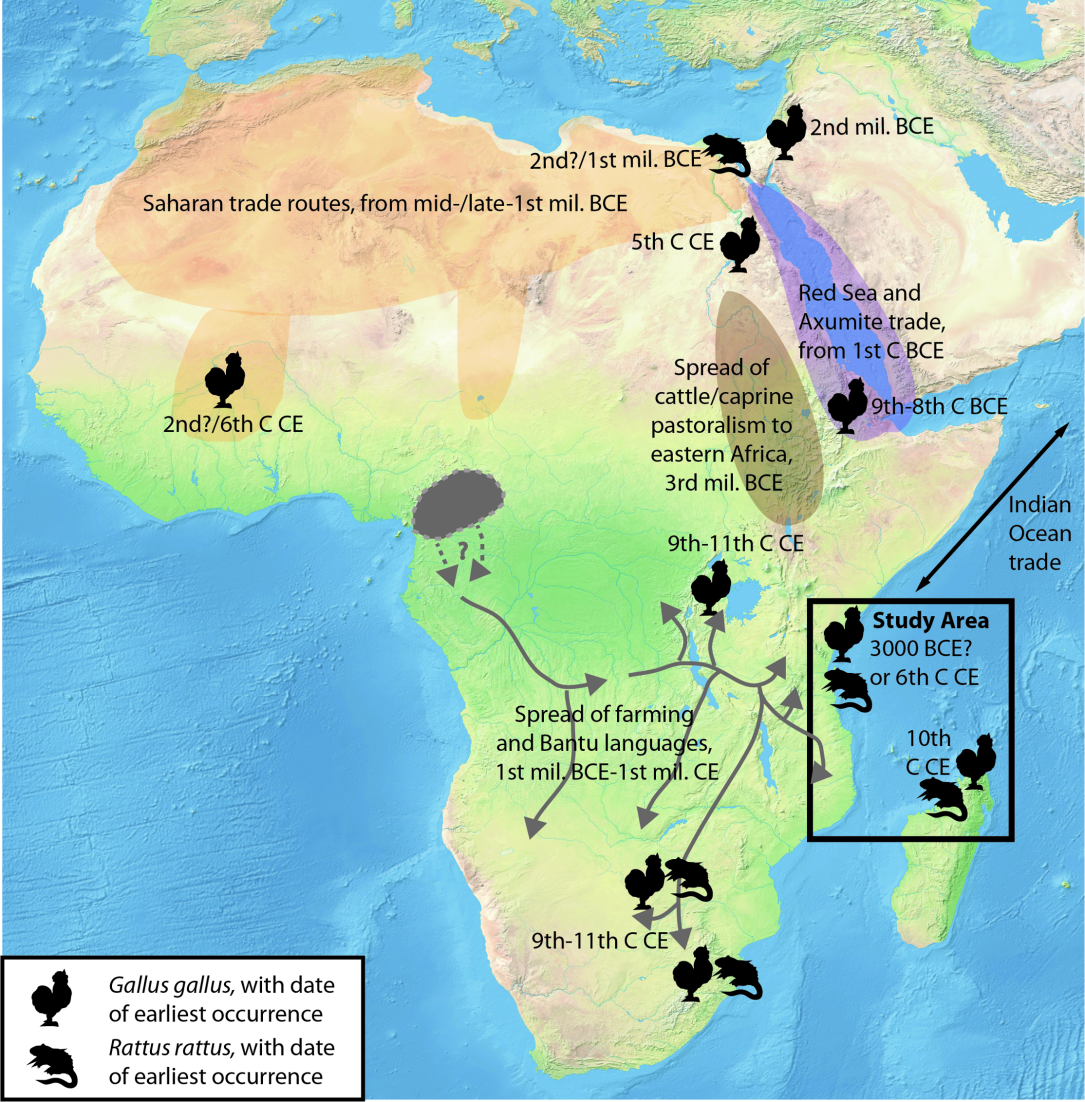
Major zones of interaction and migration. Major zones of interaction and migration on the African continent from c. 3000 BCE-1000 CE, including spreads of farming and herding, and key areas of trade. Farming and Bantu language dispersal routes follow [31]. Earliest reported dates for two Asian taxa, black rat (*Rattus rattus*) and chicken (*Gallus gallus*), are based on published data [26, 28–30, 32]. Made with Natural Earth (http://www.naturalearthdata.com).

Both models posit maritime introductions. However some Asian taxa such as domestic chicken and black rat appear in northeastern Africa substantially earlier than on the Swahili coast [24–26], so an overland introduction is theoretically possible, perhaps via mobile pastoralists or, in the case of black rat, self-dispersal is also possible. Indeed, chickens reached farming communities in western Africa via terrestrial routes, possibly as early as c. 100 BCE [27]. However, terrestrial dispersals of chicken and black rat from northeastern Africa to the Swahili coast via the Nile corridor and Rift Valley, or via the Red Sea and Horn of Africa, are not supported by faunal evidence (Fig 1). There is no evidence for these taxa among numerous late Holocene faunal assemblages from Lake Turkana in northern Kenya through the Rift Valley of eastern Africa. The only inland sites south of the Sahel with remains of these species date to the late first and early second millennium CE, postdating finds on the Swahili coast [28–30]. This suggests that maritime routes of introduction are likely. Faunal remains from coastal sites are thus critical to establishing the timing and nature of such introductions.

Issues of dating and identification have been central to debates about Asian faunal introductions to Africa, and new data and improved methods are needed to resolve the timing of arrival of individual species. For instance, elsewhere in Africa, ancient DNA analyses have challenged reports of early domestic sheep (*Ovis aries*), sparking debate about the value of biomolecular *versus* morphological bone identifications [33–35]. Here, we employ both approaches to systematically examine the arrivals of black rat and domestic chicken to the eastern African coast. Our study involved the stratigraphically controlled excavation of 16 archaeological sites, and the systematic recovery and zooarchaeological analysis of faunal remains, including microfauna. We applied biomolecular and, where possible, morphometric techniques to confirm morphological identifications of Asian fauna at these and previously excavated sites. In total, our study investigated 22 sites covering Kenya, Tanzania and its islands, the Comoros, and Madagascar. For black rat, ancient DNA (aDNA) analysis, dental morphological analysis, and Zooarchaeology by Mass Spectrometry (ZooMS) collagen fingerprinting were applied. For chicken, ZooMS was not attempted due to known taxonomic limitations within the class Aves; however, secure identification was possible through multiple lines of evidence, including classical (PCR) and high-throughput (shotgun) sequencing of ancient remains, combined with BLAST-based computational analysis. These techniques allowed us to definitively identify archaeological bone specimens to genus or species level, and shotgun sequencing furthermore enabled an assessment of ancient DNA authenticity by assessing damage patterns. Specimens confirmed as black rat or domestic chicken were then directly dated using the accelerator mass spectrometry (AMS) radiocarbon method when possible, allowing the construction of precise chronologies for their introductions.

## Materials and methods

### Permission to conduct research

All necessary permits were obtained for the described study, which complied with all relevant regulations. Permissions to conduct research were granted by the following authorities: in Kenya, the National Commission for Science, Technology, and Innovation (NCST/RRI/12/1/SS/541, NCST/RR1/12/1/SS/541/3) and National Museums of Kenya (NMK) (NMK/GVT/2, NMK/ACL/RSC/114); in Tanzania, the Commission for Science and Technology (COSTECH 2012-303-ER-2011-85) and Antiquities Division (EA.402/605/01); in Zanzibar, the Office of Chief Government Statistician (Zanzibar Research Committee) and Department of Museums and Antiquities; in the Comoros, the Centre National de Documentation et de Recherche Scientifique; in Madagascar, the Institut de Civilisations and Musée d'Art et d'Archéologie de l'Université, Antananarivo. Specimens are currently stored at the Max Planck Institute for Human History in Jena, Germany. Specimen numbers and detailed contextual information are provided in the Supporting Information.

### Archaeological sites, radiometric dating, and faunal samples

Zooarchaeological data were obtained from 22 sites in eastern Africa, 16 of which were excavated in 2010–2013 by the Sealinks Project (http://www.sealinksproject.com), the remainder in separate campaigns (S1 and S2 Tables). Sites include both cave and open-air types, and coastal, island, and hinterland locations; they date to the Later Stone Age (LSA; up to c. 600 CE), Early Iron Age (EIA; c. 100–600 CE), Middle Iron Age (MIA; c. 600–1000 CE), and/or Later Iron Age (LIA; c. 1000–1650 CE). Sealinks Project excavations followed identical procedures of single-context excavation and either dry sieving (3 mm mesh), or flotation (0.3 mm mesh) and wet sieving (1 mm mesh) of samples from each context. Other campaigns used variable excavation methods, but reported mesh sizes similarly ranging from 2–5 mm.

For Sealinks Project sites, context dates were obtained via charred seeds and charcoal at the Oxford Radiocarbon Accelerator Unit and the Waikato Radiocarbon Laboratory. Direct dates for selected bones were obtained at the latter facility. Dates were calibrated using OxCal v.4.2.4 [36], employing a mixed curve that combines the SHCal13 and IntCal13 curves at ratios of either 70:30 (Kenya, Tanzania, and offshore islands) or 80:20 (Comoros and Madagascar) to account for the effects of the intertropical convergence zone [37, 38].

Faunal remains from Sealinks Project sites were studied with reference to collections of the National Museums of Kenya (NMK) and the Zoological Research Museum Alexander Koenig in Bonn (ZFMK). Specimens that were identified as either domestic chicken (following criteria in [24]) or as chicken-sized phasianids, and those identified as either black rat or rat-sized murids, were selected for further analyses following protocols illustrated in S1 Fig. These protocols were modified over the course of the four-year study in an ongoing response to opportunities for sample export and destructive analyses, as well as analytical outcomes. For example, due to a paucity of bird remains in the 2010–2011 excavations, we were less selective in sampling these assemblages, but in subsequent field seasons we chose more confidently-identified, relatively complete specimens. For rodent remains, initial aDNA trials targeted specific contexts at relatively few sites, and focused on skeletal elements confidently attributed to black rat. In later excavation campaigns, we determined that ZooMS could be applied quickly and cost-effectively to larger samples with more diverse states of preservation. We therefore became less selective both in terms of contexts and faunal remains sampled.

### Ancient DNA analysis of bird bones

Initial molecular work on bird remains was conducted in the Durham Evolution and Ancient DNA (DEAD) Laboratory, a dedicated clean room facility. Twenty-eight samples were amplified using chicken-specific primers, which together with PCR amplification and sequencing methods are described in S1 Appendix. Sequences were visualised using Geneious Pro 5.3.4 and aligned with published *G*. *gallus* sequences [39, 40] using MAFFT v7.017 [41]. For samples that failed to produce sequences (n = 23), an attempt was made to amplify a 12S fragment. Sequences were compared against the GenBank database (http://www.ncbi.nlm.nih.gov) using a BLASTN algorithm (http://www.ncbi.nlm.nih.gov/BLAST) to search for highly similar sequences.

Due to low success rates, 19 of the originally tested specimens, and two previously untested specimens, were selected for high-throughput (shotgun) sequencing (n = 21), which has been shown to be a powerful tool for species identification, for example in archaeological dental calculus [42]. DNA extractions from bone powder were carried out in a dedicated clean room facility at the Max Planck Institute for the Science of Human History in Jena, Germany. Extraction, amplification, and sequencing methods are detailed in S1 Appendix. The resulting sequences are publicly available via the Dryad Digital Repository [43].

The resulting libraries were analysed using a novel computational technique based on a custom BLAST database that includes all 957 bird mitochondrial DNA (mtDNA) genomes available on GenBank, representing 273 genera. We used BLAST to compute an alignment score (e-value) for each genus/read combination and summarize this information across all reads in a library. Thus, while a single read may not provide enough information to allow a sample to be confidently assigned to a genus, information across multiple reads can be leveraged to provide a robust taxonomic assignment of that library. Mitochondrial DNA is ideal for this technique, first due to the availability of bird mtDNA genomes on GenBank, and second because mtDNA is on average more variable than nuclear DNA.

BLAST e-values were computed across all reads from the same sample, and were used to calculate a *p* value: the probability that a library belongs to a particular bird genus, or to a species closely related to that genus (see below). First, all mtDNA reads were re-aligned to all mtDNA genomes using BLASTN. The lowest *e*-value per genus was then extracted for each read/genus combination (in case multiple mtDNA genomes from the same genus were available in the database). Next, for each library/genus combination we computed the overall alignment score *l*_*g*_, by summing e-values for each genus over all reads in a library as:

$$l_{g}=\overset{n}{\underset{i=1}{\sum }}{log}_{10}(1-e_{i})$$

where *n* is the number of reads per library and *e* the e-value.

To eliminate poor quality alignments, we required each library’s highest *l*_*g*_ value to result from at least 20 reads alignment with *e*<10^−6^. Finally, for each best- and second-best-matching genus we computed:

$$p_{g}=\frac{10^{l_{g}}}{\sum_{i=1}^{n}10^{l_{i}}}$$

where *g* is the best- or second-best-matching genus and *n* is the number of genera (n = 273).

The resulting *p* values cannot be interpreted strictly as the probability that a library belongs to the corresponding genus, but rather as the probability that a library belongs to a species most closely related to this genus, since our database does not contain all extant bird genera. However, it does contain all *Gallus* species; furthermore, all the potential African genera that could be mistaken as *Gallus*–for example, *Numida*, *Francolinus*, or *Pternistis*–are either represented in our custom database, or have closely related genera in our database.

To confirm that our identifications derived from authentic aDNA, we used mapDamage [44] to compute 5’ C to T and 3’ G to A deamination patterns at the end of the reads. All libraries that yielded positive species identification show clear evidence of deamination, as shown in S2 Fig. To eliminate the possibility of false positives, we used a controlled experiment to test the ability of our BLASTN pipeline to retrieve the correct genus. We generated multiple “libraries” based on whole mtDNA genomes from the genus *Gallus* by randomly sampling 50 to 1000 unique sequences from a single mtDNA genome. To mimic DNA fragmentation, we randomly drew sequence lengths from a normal distribution with a mean of 40 base pairs (bp) and a standard deviation of 7 bp. We generated a total of 500 test libraries (100 replicates for each category; S3 Table) and processed these as real data. We then computed the number of false positive species identifications, which we define as cases for which the incorrect genus was obtained at a p-value >0.9. We found none, even with only 50 reads; rather, as discussed below, all our archaeological libraries have >100 reads mapping to the bird mtDNA genome database. Our approach is thus robust against false positives.

### Ancient DNA analysis of rodent bones

Twenty-three rodent bones were tested in the DEAD lab, using routine extraction techniques (S1 Appendix). Prior to analysis, all published cytochrome *b* (cyt*b*) sequences of the genus *Rattus* were aligned, and a short length of sequence was identified that can distinguish *R*. *rattus* lineages I and II (*sensu* [45]) from all others within the *R*. *rattus* complex, as well as from other common genus members, Norway rat (*R*. *norvegicus*) and Pacific rat (*R*. *exulans*). Based on this assessment, partial sequences from position 14,250–14,273 relative to EU27307 [46] were amplified in a PCR reaction and either Sanger sequenced and/or *de novo* Pyrosequenced, following methods described in S1 Appendix. The ancient DNA sequences were aligned with the *Rattus* species dataset and their identifications were determined by comparison of SNPs between target and known sequences. Where sequences deviated from expected variation (on visual inspection), a sequence search was performed in the GenBank nucleotide database using BLASTN.

### Screening of rodent crania by tooth morphology

Tooth morphology was used to determine which rodent specimens were good candidates for ZooMS collagen fingerprinting, for 17 well-preserved cranial specimens selected from four sites. Reference material for eastern African native rodents was not available at the time of analysis, so comparisons could only be made with island Southeast Asian and invasive species, obtained from the Montpellier CEROPATH project (S4 Table). Identifications were considered preliminary, to be confirmed by ZooMS.

Geometric morphometric data were gathered from photographs of the first mandibular molar. Shape was quantified using a combination of five fixed and 82 sliding semi-landmarks (S3 Fig). Size was quantified as centroid size. Both shape and form (shape+log centroid size) were then used to define morphological variability in the reference material. Archaeological specimens were assigned to a group by applying a discriminant analysis and comparing the unknown specimens to the group distributions using posterior probabilities. Our expectation was that *R*. *rattus* specimens would be more likely to match a species of that genus, whereas native African species would group with other genera.

### ZooMS analyses of rodent bones

The 17 cranial specimens described above, and an additional 215 mainly postcranial specimens, were analysed via Zooarchaeology by Mass Spectrometry (ZooMS) at the University of Manchester. This sample included one specimen that had failed aDNA testing; however, due to the small size of the remains, there were no other specimens to which both techniques were applied.

Reference material was acquired from the University of Manchester, the ZFMK, the Muséum national d'Histoire naturelle (MNHN) in Paris, and the Royal Belgian Institute for Natural Sciences (RBINS) in Brussels (S5 Table). Collagen fingerprints were obtained from reference samples following established methods [47], described in S1 Appendix. These modern specimens show distinct collagen fingerprints, as represented by peak *m/z* values. As shown in S4 Fig, *R*. *rattus* may be distinguished from *R*. *exulans* (Pacific rat) and *R*. *norvegicus* (brown rat) [48]. In order to potentially identify other rodent taxa in the archaeological assemblages, we also obtained collagen fingerprints from six murid genera common to eastern African landscapes: *Aethomys*, *Gerbilliscus*, *Mastomys*, *Mus*, *Otomys*, and *Thallomys*. These are distinctive among themselves and from *Rattus*, as shown in S5 Fig. Collagen fingerprints obtained from archaeological samples, following identical methods to that of the modern reference material, were categorized into groups that yielded the same peptide markers, and were visually compared against the reference spectra to attempt to infer the most closely related taxon. Identifications were considered confident when all peaks within the archaeological specimen were observed in the modern reference spectra, but not vice versa (since modern samples typically contain more peptides).

## Results

### Zooarchaeological and dental morphological analyses

**Fig 2:**
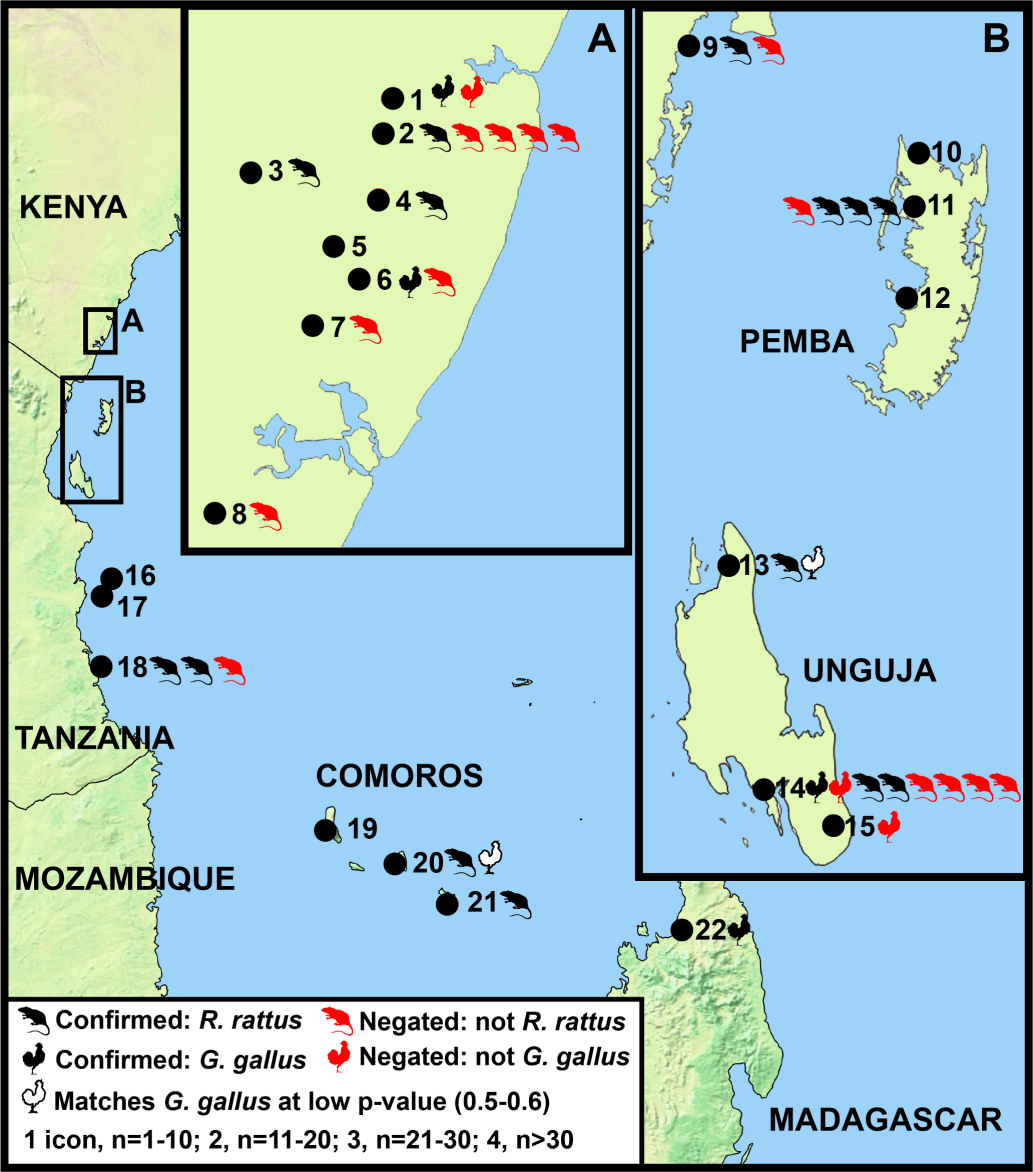
Results of biomolecular analyses. Results of biomolecular confirmation or negation of domestic chicken (*Gallus gallus*) and black rat (*Rattus rattus*) remains identified via zooarchaeological analyses. Sites: 1, Mulungu wa Mawe; 2. Panga ya Saidi; 3, Mtsengo; 4, Panga ya Mwandzumari; 5, Kwa Kipoko; 6, Panga ya Mizigo; 7, Mbuyuni; 8, Chombo; 9, Vumba Kuu; 10, Pango la Watoro; 11, Makangale Cave; 12, Ras Mkumbuu; 13, Fukuchani; 14, Unguja Ukuu; 15, Kuumbi Cave; 16, Juani Primary School; 17, Ukunju Cave; 18, Songo Mnara; 19, Nyamawi; 20, Sima; 21, Dembeni; 22, Mahilaka. Main figure made with Natural Earth (http://www.naturalearthdata.com); inset maps were hand-drawn.

Of the 20,636 specimens (Number of Identified Specimens, NISP) analysed at the Sealinks Project sites, 7,324 were identified to order or lower taxonomic levels, based on morphological criteria. This assemblage included 52 specimens identified as domestic chicken or as chicken-sized phasianids, and 444 specimens identified as black rat or as rat-sized murids. Six sites produced no specimens identified as possible Asian taxa (Table 1, Fig 2). At eight sites, the frequencies of possible black rat and/or possible domestic chicken were low (<1% to 3% of NISP).

**Table 1:** Summary of results. Zooarchaeological and biomolecular results for the sites studied, with earliest dates for fauna confirmed via biomolecular analysis.

			Domestic chicken						Black rat								
			NISP^1^		aDNA			Earliest date CE (bold = direct)	NISP^1^		aDNA			ZooMS			Earliest date CE (bold = direct)
Site	Type	Total NISP^1^	C	U	+ (*p*)^2^	- (*p*)^2^	F		C	U	+	-	F	+	-	F	
MM^3^	Cave	N/A	2	6	4	1	3	**1695–1930**									
PYS	Cave	2259	1	2			1			141		2	1	1	88	49	8^th^-17^th^ C
MTSE^4^	Open	1825	38						2					1			**1435–1490**
SC^3^	Cave	N/A								2	1		1				**1495–1640**
KK	Open	2															
PMZ	Cave	566	1	3	1			modern		3					1	1	
MBYN^4^	Open	990	16						3			1	1				
CHO^4^	Open	718	6						3			1	1		1		
VMB^4^	Open	548	171						18		1				4		15^th^ C
PLW	Cave	89															
PK	Cave	1330							242					29	1		**680–865**
RM	Open	11															
FK	Open	273		1	1 (.54)			7th-8th C	1	1	1			1			≥7^th^-8^th^ C
UU^4^	Open	254							1				1	1			**421–535**
UU	Open	758	3	17	1 (1.0)	1 (.99)	4	8th-10th C	3	50				13	37		**580–645**
KC	Cave	1874		9		3 (.99)	3										
JS	Open	81															
PU	Cave	10															
SM^4^	Open	304	80						30		9	1	1	2			**1320–1425**
NMW	Open	3															
SMA	Open	68	2		1 (0.5)		1	7th-10th C		1				1			7^th^-10^th^ C
DMB^4^	Open	417	>2						11					1			8^th^-10^th^ C
MHLK^3^	Open	N/A		5	1 (.99)		4	10th-13th C									

Sites listed in north to south order; see S1 and S2 Tables for site codes. C, confident; U, uncertain; +, positively confirmed as chicken/black rat; -, identification negated; F, failed to produce readable data; *p*, probability.

^1^ Based on zooarchaeological analysis; total NISP (Number of Identified Specimens) = nonhuman tetrapods identified to order or lower.

^2^ *p*-values applicable only where all mtDNA genomes were used; see text for details.

^3^ Specimens were selected during excavation and no zooarchaeological analysis was conducted; total NISP not available.

^4^ Sites excavated prior to the current project; NISP values from publications (S2 Table); only black rat was analyzed.

Two sites stood apart from this trend: Panga ya Saidi had very high numbers of rat-sized murids, identified at a low level of confidence (8% NISP), while Makangale Cave revealed exceptionally high numbers of black rat remains, identified at a high level of confidence (18% NISP). Six cranial specimens from Panga ya Saidi, and seven from Makangale Cave, were selected for dental morphological analysis. In addition, one specimen from Panga ya Mizigo and three from Unguja Ukuu were chosen on the basis of preservation and contextual associations. All of the cranial specimens from Makangale Cave and Unguja Ukuu closely matched the reference specimens for the genus *Rattus*, coinciding with their high-confidence identifications using traditional zooarchaeological methods. No specimens from Panga ya Saidi and Panga ya Mizigo–identified more generally as rat-sized murids–did so. ZooMS collagen fingerprinting confirmed these attributions to *Rattus* and non-*Rattus* groups (see below).

### Confirmation and negation of *Gallus gallus* identifications

Specimens morphologically identified as chicken or chicken-sized phasianids were selected for aDNA analysis following protocols outlined in S1 Fig. High failure rates during initial tests indicated low amounts of preserved endogenous DNA: of 28 specimens amplified using chicken-specific primers, only five (18%) generated bands of the expected size for chicken; of 23 specimens for which the 12S fragment was amplified, all failed except one, which matched hornbill (*Bycanistes brevis*). Details of these results can be found in S6 Table.

Success rates increased substantially for the 21 specimens to which shotgun sequencing and the custom BLAST approach were applied, as shown in S6 Table: while 13 failures (62%) suggest potential human contamination in the shotgun libraries, eight samples (38%) contained DNA attributed to Galliformes. S7 Table provides additional detail on the total number of reads and results of alignments in the BLAST analysis; for archaeological specimens, all libraries have >100 reads that map to the custom database. Among specimens attributed to Galliformes, matches were made to *Gallus*, and to *Numida* (guinea fowl, native to eastern Africa). Further matches were made to four Asian phasianid genera (all members of the order Galliformes): *Arborophila*, *Bambusicola*, *Polyplectron*, and *Syrmaticus*. The most likely explanation is not that these are Asian imports, but rather that these bones belong to native African genera (not yet sequenced) that are more closely related to Asian genera than to other African genera. There are a number of African phasianid genera not in the GenBank database–for example *Francolinus* and *Pternistis*–that are potential candidates.

Together, multiple aDNA methods confirmed seven chicken specimens at two cave sites in the coastal hinterland (Mulungu wa Mawe and Panga ya Mizigo), and two open-air sites on Zanzibar and Madagascar (Unguja Ukuu and Mahilaka, respectively) (Table 1, Fig 2). Two additional specimens had more ambiguous results, since p-values were comparable to those obtained for Asian phasianids. This was the case at Fukuchani on Zanzibar, where similar p-values were obtained for *Gallus* (*p* = 0.54) and *Bambusicola* (*p* = 0.46), and also at Sima in the Comoros, with similar values for *Gallus (p* = 0.5) and *Arborophila* (*p* = 0.46). While it is not possible to infer with confidence whether or not these bones belong to *Gallus*, such similar probabilities for two genera could stem from the fact that these bones belonged to a genus equally close to both (an outgroup). The aDNA analysis did however confidently negate some morphological identifications. One specimen identified as likely *Gallus* was found to match *Numida*. Four specimens identified as either Galliformes or likely Galliformes most closely matched other members of this order, not *Gallus*. A specimen identified only as “bird” was shown to not be a phasianid at all, but rather *Bycanistes brevis*.

### Chronology and distribution of confirmed *Gallus gallus* specimens

**Fig 3:**
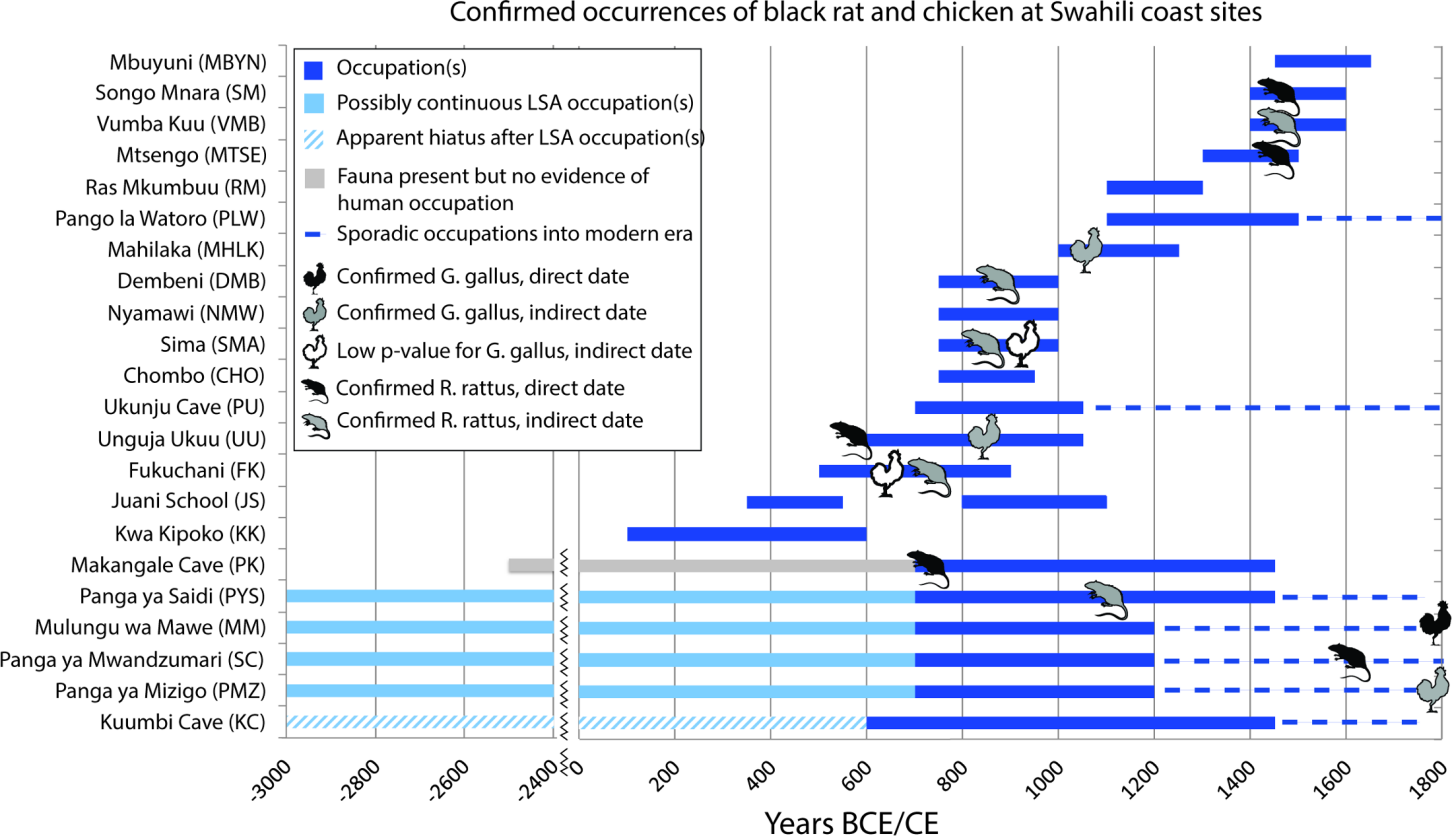
Chronology of occurrences of non-native species. Approximate years of occupation at sites in the present study, with earliest confirmed occurrences of domestic chicken (*Gallus gallus*) and black rat (*Rattus rattus*).

None of the confirmed chicken specimens are of great antiquity, as shown by Fig 3, with supporting radiocarbon dates presented in S6 Table. The only secure, pre-modern contexts bearing confirmed chicken remains are found at Unguja Ukuu and Mahilaka, both open-air port sites whose occupants were engaged in maritime trade. AMS dates on charred crop seeds in associated contexts at these sites suggest that chicken arrived on Zanzibar by the 7^th^-8^th^ century CE, and Madagascar by the 11^th^-13^th^ century CE. Meanwhile, confirmed specimens from mainland hinterland cave sites were very recent; two confirmed chicken bones from Mulungu wa Mawe were directly dated to only the 18^th^-20^th^ centuries CE, while the one from nearby Panga ya Mizigo is suspected to have a similarly late age, based on its stratigraphic position. Our results thus suggest a late first millennium CE island arrival for chicken at port sites. In contrast, chicken is found only in disturbed and/or recent contexts at cave sites in the coastal hinterland. Importantly, we failed to replicate prior reports [17] of chicken at up to 5% of NISP at Kuumbi Cave on Zanzibar [49, 50], where the only possible “chicken” specimen in the Sealinks assemblage (<1% NISP) proved to be guinea fowl.

### Confirmation and negation of *Rattus rattus* identifications

The rodent dataset is far larger than the bird dataset and exhibited higher success rates: of 23 rodent specimens amplified for aDNA analysis, 17 (74%) produced readable sequences, and 12 of these matched black rat; of the 232 rodent specimens analysed by ZooMS, collagen was successfully extracted and fingerprinted from 182 (78%), 50 of which matched black rat. These results are presented in Table 1, with greater detail in S8 Table. Although these success rates appear similar, they are not directly comparable, since the aDNA and ZooMS datasets are distinct: most aDNA-tested specimens came from 2^nd^ millennium CE sites and were selected based on expected preservation; by contrast, the much larger ZooMS set includes specimens from older contexts and/or appearing poorly preserved. Just one specimen had enough tissue remaining after aDNA extraction to enable application of both methods: it failed to produce readable aDNA but succeeded with ZooMS.

Collagen fingerprints obtained from the 182 archaeological specimens appeared to form seven groups, shown in S6 Fig and S7 Fig. These groups are based on differences in peak *m/z* values, representing the masses of peptides that differ between species [51]. These were matched with the closest reference taxa for which the collagen fingerprints yielded taxonomically hierarchical information. The spectrum from the modern *R*. *rattus* reference specimen (S4 Fig, top) identically matched those obtained from one of the groups of archaeological samples, an example of which is illustrated in S6 Fig, bottom. Another group of archaeological samples (Group 2; an example of which is illustrated in S6 Fig, top) most closely matched the reference specimen of *Gerbilliscus validus* (S5 Fig, B, top), however one consistently different marker (*m/z* 1069.7–1095.7) was observed. In this case, the archaeological specimens most likely derive from a close relative, but the exact species could not be confirmed due to current limitations in collagen fingerprinting of reference material for African rodents. The same is true of another group of archaeological specimens (Group 1), which closely matched *Mastomys*. Four additional groups (Groups 3–6) observed in the archaeological samples could not be identified, due to the same limitations. All that can be said securely is that these groups are not *Rattus*, *Gerbilliscus*, or *Mastomys*.

The results of the tooth morphology analysis, described above, agreed with those of ZooMS for all 17 specimens subjected to both analyses. Specimens morphologically identified as *Rattus* from Makangale Cave (n = 7) and Unguja Ukuu (n = 3) were confirmed as *Rattus* via ZooMS, whereas those that were morphologically a poor match for *Rattus* were found via ZooMS to be most similar to *Mastomys* (Panga ya Saidi, n = 6) or to *Gerbilliscus* (Panga ya Mizigo, n = 1).

### Chronology and distribution of confirmed *Rattus rattus* specimens

The geographic distribution of black rat (Fig 2) and the chronology of its appearance (Fig 3; supporting radiocarbon dates in S8 Table) show similar patterns to those observed for domestic chicken. Confirmed black rats in the coastal hinterland appear quite late: one specimen at the open-air site of Mtsengo was directly dated to the 15^th^ century CE, and two specimens from the cave site of Panga ya Mwandzumari were directly dated to the 16^th^-18^th^ centuries CE. Most rodents from hinterland assemblages are not black rat, but rather local wild rodents. For example, the majority of murid remains tested at Panga ya Saidi (87 out of 141) most closely resembled *Mastomys* or *Gerbilliscus*, and the only confirmed black rat specimen came from a near-surface context; the remainder belong to unidentified murid genera. *Gerbilliscus* was also identified as the closest match to specimens at the cave site of Panga ya Mizigo and the open-air site of Chombo. There is no evidence in our dataset for black rat at hinterland sites prior to the 15^th^ century CE, regardless of whether the sites are open-air or in caves.

On the coast and islands, by contrast, black rat was confirmed in secure contexts at multiple sites, including Vumba Kuu (n = 1) on the southern Kenyan coast; Songo Mnara (n = 11) in the Kilwa archipelago; Unguja Ukuu (n = 14) and Fukuchani (n = 2) on Zanzibar; Makangale Cave (n = 29) on Pemba; and Sima (n = 1) and Dembeni (n = 1) in the Comoros. Not all rodent remains at these sites belonged to black rat: specimens closely resembling *Mastomys*, *Gerbilliscus*, or other non-*Rattus* groups were also documented. However confirmed rats are relatively abundant, especially at Makangale Cave. This site produced the largest number of morphologically-identified black rats in this study (NISP = 242); an identification was confirmed via ZooMS collagen fingerprinting for all but one of the 30 specimens selected for further analyses. This is the only island cave site, out of the four included in our study, with confirmed black rat.

Three direct dates obtained on black rat bones at Unguja Ukuu suggest that the introduction of *R*. *rattus* to island eastern Africa could have been as early as the 5^th^ century CE (S8 Table). However, the specimen with the earliest date had a low gelatin yield, and it is also possible that a marine diet for black rats could create a reservoir effect impacting some or all specimens dated in this study, particularly on coastal sites where zooarchaeological analyses often indicate human diets with a significant marine component [52]. A more conservative approach is to suggest that black rat is present by the 7^th^-8^th^ centuries CE at Unguja Ukuu, based on a Bayesian model constructed for the site from 31 dates on seeds and charcoal [7]. Two of the black rat specimens from Makangale Cave on nearby Pemba were directly dated to the 7^th^-9^th^ centuries CE, coinciding with associated shell and charcoal dates. On present evidence, we conclude that black rats, like chickens, were introduced to the islands by the mid-first millennium CE; however in the coastal hinterland, these taxa are found only in disturbed or relatively late contexts.

## Discussion

While the Columbian Exchange is documented by a wealth of historical materials, our understanding of prehistoric long-distance biological exchanges is limited to what can be garnered from archaeological proxies, including the remains of domestic and commensal animal species. Archaeologists have long debated the timing, mechanisms, and social contexts of the arrivals of Asian taxa to eastern Africa, with competing models advocating introductions separated by several millennia. An early arrival of Asian species, possibly c. 3000 BCE, has radical implications for widely accepted models for the spread of food production in sub-Saharan Africa. A less contested later arrival, by the mid-first millennium CE, is however at odds with Classical-era texts attesting to well-established maritime trade several centuries earlier.

Our combined datasets offer no evidence for extremely early introductions of Asian species. Instead, all confirmed and reliably dated specimens of black rat and domestic chicken date to the second half of the first millennium or later. The study provides support for a maritime introduction of both species, since the earliest specimens are at island sites, particularly open-air settlements with associated evidence for Indian Ocean trade contacts. Our study also suggests that black rat and domestic chicken arrive later at mainland sites, and, with the exception of rats at Makangale Cave on Pemba, at cave sites as well. In contrast with the island sites, these localities all show later integration into the kinds of early trade networks that linked eastern Africa to the Indian Ocean world.

Taken together, our data provide support for an arrival of Asian taxa at port sites on islands by the 7^th^-8^th^ centuries CE (Fig 2). Domestic chicken is documented on Zanzibar by the 7^th^-8^th^ centuries CE, and may be present on the Comoros by the 8^th^-10^th^ centuries. Black rat may appear still earlier on Zanzibar, based on a single direct date of 421–535 CE at Unguja Ukuu, but isotopic research is required to rule out the potential reservoir effects in case of a marine diet for this and other directly-dated black rat specimens. A more conservative estimate, based on associated directly-dated crop remains, is to suggest that black rat appears at Unguja Ukuu and Fukuchani by the 7^th^-8^th^ centuries CE, coincident with the appearance of domestic chicken in our dataset. This range also coincides with that for black rat remains at Makangale Cave on Pemba, where this taxon is unusually abundant. In our dataset, neither black rat nor domestic chicken appears at mainland sites until the second millennium CE.

Our investigations at six cave sites spanning the LSA to MIA offered no evidence for Asian taxa in the millennia suggested by the early model of Asian introductions (Fig 3). At Kuumbi Cave, where domestic chicken was previously reported at 5% of NISP [17], new excavations produced just one possible domestic chicken specimen [49, 50], which was then demonstrated via aDNA analysis to be local guinea fowl. At two open-air EIA sites, Juani School and Kwa Kipoko, not a single possible Asian faunal specimen was identified. While future excavations and analyses may produce Asian fauna in pre-MIA sites, much earlier introductions seem unlikely, given our dataset’s geographic and chronological coverage.

Late arrivals of domestic chicken and black rat suggest that engagement of the Swahili coast with the Indian Ocean world began in earnest only in the Medieval period (7^th^-15^th^ centuries CE) or shortly before. This finding agrees with other archaeological datasets such as imported ceramics and glass beads, which only began to arrive in significant quantities in the same era, together with Asian crops [32, 53]. Like these other data classes, faunal remains currently do not support accounts of regular trade addressed in Classical-era documents. While biological exchange in the Indian Ocean, including exchange between the Indian subcontinent and northeastern Africa via the Red Sea, certainly predates the Medieval period, our findings fit with an emerging picture of Medieval maritime intensification [54, 55] that seems to have resulted in more frequent transference of species by sea, and a more expansive sphere of biological exchange.

Our findings also contribute to understanding the origins of African food production systems by supporting multiple introductions to the continent of domestic chicken, an economically and ritually significant species today [56]. Domestic chickens arriving in western Africa as early as c. 100 CE [27] were likely introduced via Egypt and/or the Red Sea and Horn of Africa, without implicating eastern Africa. Late first-millennium CE appearances of domestic chicken in central and southern African sites [28–30] postdate our findings in Zanzibar and the Comoros. Domestic chicken likely spread to these areas via mainland eastern Africa, and/or via additional introductions along the coast. Modern phylogenetic analyses (e.g., [57]) are beginning to shed light on these processes and will be enriched by aDNA analyses of securely-dated faunal remains.

The ecological implications of our findings also deserve consideration and further research. Introduced rats, for example, are clearly associated with destructive impacts, particularly on islands, as seen throughout the Pacific and Mediterranean regions [58–60]. In the present study, the greatest number of black rat remains was found in a Pemba Island cave. Pemba has been isolated from the mainland since at least the Pliocene [61] and is depauperate in endemic fauna [62]. Low faunal diversity and an absence of predators on islands offer optimal conditions for rapid spread of black rat, as shown elsewhere [59, 63, 64], and may well explain its archaeological abundance on Pemba. The effects of the black rat’s introduction on Pemba and in other eastern African contexts deserve investigation. In addition to habitat destruction, black rats cause economic losses to farmers [59]. Attraction to grain stores likely explains their prevalence at the relatively urban agricultural site of Unguja Ukuu. The coincident appearance of domestic cats (*Felis catus*) at this port site [65] may well be connected to the pest problem posed by black rats, both on ships and on land.

On a more speculative note, recent research has focused on the origins and spread of the plague bacterium, *Yersinia pestis*, for which black rats are perhaps the most infamous though not the only vector [59]. Plague may well have traveled with black rats on ships to eastern Africa during or following the Justinian Plague of 541–542 CE, which spread from East Asia [66] westward to the Mediterranean basin, possibly via the Red Sea [67]. Subsequent plague outbreaks have gone unstudied in the western Indian Ocean, with the first eastern African instance of the disease only being recorded in colonial-era Uganda [68]. Research may reveal earlier appearances of plague, a disease of particular contemporary interest in Madagascar and eastern Africa, where it claims more victims today than anywhere else in the world [69]. Better quality archaeological data, obtained through more rigorous application of available dating and biomolecular techniques, are key to revealing the historical contexts of this and other aspects of biological exchange across the Indian Ocean.

Biomolecular sources of information provide important insights into ancient processes that are difficult or impossible to address using traditional archaeological methods. Our results demonstrate that such methods are applicable even in hot, tropical contexts where ancient DNA preservation is not optimal. While poor preservation likely accounts for our low aDNA success rates for bird specimens, we found that twice as many specimens provided readable sequences once shotgun sequencing was employed, using a novel analytical approach based on a custom-built BLAST database. On the other hand, limitations of aDNA application do persist in the tropics, and our findings also suggest that ancient proteins offer a useful solution. ZooMS collagen fingerprinting allows for quick and affordable confirmation of species in a wide range of preservation states, and as such will become increasingly applicable to archaeological problems in sub-Saharan Africa, and elsewhere in the tropics.

## Supporting information

S1 Appendix Details of methods used in ancient DNA (aDNA) and Zooarchaeology by Mass Spectrometry (ZooMS) collagen fingerprinting analyses. (DOCX)

S1 Table Sites excavated by the Sealinks Project. (DOCX)

S2 Table Previously excavated sites included in the present analysis. (DOCX)

S3 Table Results of experimental study of false positives. Incorrect genus identifications resulting from 500 test "libraries" obtained from whole mtDNA genomes of the genus *Gallus*. See text for explanation of experimental method.
(DOCX)

S4 Table Reference specimens for analysis of tooth morphology. (DOCX)

S5 Table Reference specimens for ZooMS collagen fingerprinting. (DOCX)

S6 Table Detailed results for bird specimens. Results of multiple ancient DNA analyses, with radiocarbon dates where available. Sites ordered from north to south.
(DOCX)

S7 Table Total reads used in the BLAST analysis and results of Burrows-Wheeler Alignments (BWA). (DOCX)

S8 Table Detailed results for rodent specimens. Results of ancient DNA analysis, ZooMS collagen fingerprinting, and tooth morphology, with radiocarbon dates where available. Sites ordered from north to south.
(DOCX)

S1 Fig Decision tree illustrating research protocols. Tree illustrates the selection of faunal samples, the order in which specific analyses were applied to each subsample, and result.
(TIF)

S2 Fig mapDamage analysis of deanimation patterns in bird specimens. For each of the sequenced specimens (specimen numbers indicated by JK0000), mapDamage analysis illustrates C to T (red) and G to A (blue) frequencies of mis-incorporation at 3’ and 5’ ends.
(PDF)

S3 Fig Landmarks used in dental analysis. *R*. *exulans* tooth in occlusal view with simplified diagram to the right. The fixed landmarks are illustrated by large blue circles, sliding semi-landmarks by small red circles. The boundaries of the cusps and the stylids (small flat or saddle like surfaces joining cusps) are difficult to precisely identify, but have been illustrated in the diagram for clarity.
(TIF)

S4 Fig Spectra from modern *Rattus* taxa. MALDI peptide mass fingerprint spectra of collagen tryptic digests from the reference bone material of *Rattus rattus* (top), *Rattus norvegicus* (middle) and *Rattus exulans* (bottom).
(TIF)

S5 Fig Spectra from modern rodent genera other than *Rattus*. **A**: MALDI peptide mass fingerprint spectra of collagen tryptic digests from the reference bone material of *Aethomys kaiseri* (top), *Mastomys coucha* (middle) and *Mus minutoides* (bottom). **B**: MALDI peptide mass fingerprint spectra of collagen tryptic digests from the reference bone material of *Gerbilliscus validus* (top), *Otomys tropicalis* (middle) and *Thallomys paedulcus* (bottom).
(TIF)

S6 Fig Example of MALDI peptide mass fingerprint spectra in archaeological samples. Example of MALDI peptide mass fingerprint spectra of collagen tryptic digests from the archaeological samples studied, showing the three most commonly identified types: *Rattus rattus* (bottom); Group 1 (middle), which most closely resembles *Mastomys;* and Group 2 (top), which most closely resembles *Gerbilliscus*.
(TIF)

S7 Fig Spectra from unknown taxa in archaeological samples. MALDI peptide mass fingerprint spectra of collagen tryptic digests from archaeological specimens that form groups of unknown taxa.
(TIF)
