# Correction: Reconstructing Asian faunal introductions to eastern Africa from multi-proxy biomolecular and archaeological datasets

**DOI:** 10.1371/journal.pone.0190336

**Published:** 2017-12-20

**Authors:** The PLOS ONE Staff

The twelfth author’s name is spelled incorrectly. The correct name is: Eréndira M. Quintana Morales. The publisher apologizes for the error.
