# Supplementary material for: Reconstructing Asian faunal introductions to eastern Africa from multi-proxy biomolecular and archaeological datasets
Source: PLoS One. 2017 Aug 17;12(8):e0182565. doi: 10.1371/journal.pone.0182565 (PMC5560628; doi:10.1371/journal.pone.0182565)
Supplement: S1 Appendix — (DOCX) [file pone.0182565.s001.docx]

**S1 Appendix: Details of biomolecular methods**

**A) aDNA amplification of bird bones using chicken-specific primers**

Molecular work was conducted in the Durham Evolution and Ancient DNA (DEAD) Lab, an isolated, dedicated ancient DNA laboratory at Durham University. Twenty-eight bird bones were sampled. Approximately 1mm of the exterior surface was removed using a Dremel hand-drill, after which each sample was ground to fine powder in a sterilized stainless steel canister using a Sartorius microdismembrator. Samples were incubated overnight at 50°C in a solution containing 1.7ml of 0.5M EDTA (pH8), 0.2ml of 10mM TRIS-HCl (pH8), 0.1ml of SDS (1%w/v) and 20µl of Proteinase K (20mg/ml). The QIAquick PCR purification kit (Qiagen) was used following the manufacturer's instructions.

The chicken-specific primer pair GG144F/GG387R was used to test for *Gallus gallus*. The forward primer GG144F (5'-ACCCATTATATGTATACGGGCATTAA-3') and the reverse primer GG387R (5'-CGAGCATAACCAAATGGGTTAGA-3') were used to amplify a 201bp fragment (excluding primers) of the mitochondrial control region. The PCR amplifications were performed in 25µl containing 2µl of extract, 0.76x PCR Gold Buffer, 1.89mM MgCl_2,_ 1.04U 04U *Taq* DNA polymerase (AmpliTaq Gold), 0.18mM dNTP and 0.75µM of each primer. 2.5µl of BSA (25mg/ml) was added. The PCR thermal cycling reactions comprised of a 90s initial denaturation at 94°C, followed by 50 cycles of 30s denaturation at 94°C, 30s annealing at 54°C, an extension of 30s at 72°C, and a 10min final extension at 72°C. Amplifications were visualized on a 0.5x agarose gel. Sequencing was performed in both directions on an ABI 3730 sequencer in the DEAD Lab; sequences were visualized using Geneious Pro 5.3.4 and were aligned with previously published *G. gallus* sequences (1, 2) using MAFFT v7.017 (3), followed by visual confirmation.

For samples that failed to produce DNA sequences using the chicken-specific primer, an attempt was subsequently made to amplify a 12S fragment. The forward primer 12Sa (5'-GGGATTAGATACCCCACTAT-3') and the reverse primer 12SEr (5'-CGATTATAGAACAGGCTCCTC-3') were used to amplify a 105bp fragment excluding primers. PCR amplifications were performed in a 25µl reaction mix identical to that described above. Again, 2.5µl of BSA (25mg/ml) was added. The PCR thermal cycling reactions consisted of a 90s initial denaturation step at 94°C, followed by 50 cycles of 45s denaturation at 94°C, 45s annealing at 48°C, a 45s extension step at 72°C, and a 10m final extension step at 72°C. The amplified extracts were sequenced as described above, and the obtained 12S sequences were compared against the GenBank database using a BLAST algorithm. All sequences except one were identified as 'human’, meaning not enough endogenous DNA was present within the bones for it to be recovered. The remaining sample matched the silvery-cheeked hornbill, *Bycanistes brevis* (GenBank number HM640210).

**B) mtDNA BLAST approach**

Given the low success rates of the above approaches, 19 of the tested specimens, and an additional two previously untested specimens, were selected for high-throughput (shotgun) sequencing. DNA extractions from 50-60 mg bone powder for each sample were carried out in clean room facilities dedicated to ancient DNA work at the Max Planck Institute for the Science of Human History in Jena, Germany, using an established silica purification protocol (4) with the following modifications: the Zymo-Spin V columns (Zymo Research) were UV irradiated for 30min and the total elution volume was raised to 100 µl. A 20 µl aliquot of each DNA extract was converted into double-stranded Illumina libraries (5). Sample-specific indexes were added to both library adapters via amplification (6) to obtain double-indexed libraries. Extraction and library blanks were treated accordingly. A second amplification was performed for all indexed libraries in 100µl reactions containing 5µl library template, 4 units AccuPrime Taq DNA Polymerase High Fidelity (Invitrogen), 1 unit 10X AccuPrime buffer (containing dNTPs) and 0.3 µM IS5 and IS6 primers (5). The following thermal profile was used: 2min initial denaturation at 94°C, followed by 4 to 17 cycles consisting of 30s denaturation at 94°C, 30s annealing at 60°C, a 2min extension at 68°C and a 5min final extension at 68°C. Purification of the amplified products were carried out using MinElute spin columns (Qiagen) according to the manufacturer’s protocol. The amplified indexed libraries were quantified using an Agilent Bioanalyzer DNA 1000 Chip, subsequently diluted to 10nM and pooled in equimolar amounts for sequencing. The sequencing was performed on an Illumina NextSeq 500 platform via 2*150+8+8 cycles using the NextSeq High Output reagent kit v1 and the manufacturer’s protocol for multiplex sequencing.

For each library, we removed adapter sequences and merged overlapping pairs using AdapterRemoval (7). Merged and clipped reads were then aligned to the chicken genome (Galgal4), the human genome (HG19), the Japanese quail genome (*Coturnix japonica*), and the mitochondrial genomes of all 957 birds available on GenBank using BWA(8) with ancient DNA settings(9). We removed duplicate reads using Picard Tools MarkDuplicates (http://broadinstitute.github.io/picard/).

In order to compute the probability that a bone (*i.e.* a library) belongs to the genus *Gallus*, we undertook a custom blast-based approach. We first built a “blastable” database based on all (957) mtDNA genomes of birds available on GenBank. These represent 273 genera (with 95 mtDNA genomes belonging to the genus *Gallus*). Before blasting reads we aligned these to the 957 mtDNA genomes using BWA (8) to avoid blasting non-endogenous or autosomal reads as well as to increase computational efficiency. Thereafter, to estimate the probability that a given library belongs to *Gallus* we took the following steps:

1. Re-align all mtDNA reads to all the mtDNA genomes using BLASTN.
2. Extract the *e*-value for each read/genus combination (lowest e-value per genus).
3. For each library/genus combination we computed the overall alignment score *l_g_* :

$$l_{g}=\sum_{i=1}^{n} {log}_{10}(1-e_{i})$$

Where *n* is the number of reads per library and *e* the value.

1. In order to filter out bad quality alignments, we required that each library’s highest *l_g_* value was the result of at least 20 reads alignment with *e*<10e^-6^.
2. For each best and second best genus we computed:

$$p_{g}= \frac{{10}^{l_{g}}}{\sum_{i=1}^{n} {10}^{l_{i}}}$$

where *g* is the best or second best genus and *n* is the number of genera (n=273).

It is important to note that the resulting *p* value is not the probability that a given library belongs to the corresponding genus but rather the probability that it belongs to a species that is most closely related to this genus. Indeed, our set of 273 genera does not contain all extant bird genera. Nevertheless, given that our set contains all *Gallus* species, we believe that this is a very conservative approximation of whether a bone belongs to *Gallus* or not. This is because all potential African species that could be mistaken as *Gallus* have closely related genera in our blast database (i.e. *Numida*). Thus, if a bone does not belong to *Gallus* we expect that it would yield a much higher *p* value for a species that is most closely related to it and a very low *p* value for *Gallus*. Lastly, if a library has limited information (short reads, low endogenous content) it will yield equivalently low *p* value for many genera.

**C) Sanger and Pyro-sequencing to identify *Rattus rattus***

Analyses took place in the DEAD Lab at Durham, described above. All published cytochrome *b* (cyt*b*) sequences of the genus *Rattus* were aligned and a short length of sequence was identified that can distinguish *Rattus rattus* lineages I and II (*sensu* (10)) from all other taxa within the *R. rattus* complex, as well as from other common members of the genus (the brown rat *R. norvegicus* and the Pacific rat *R. exulans*). Partial sequences from position 14,250-14,273 relative to EU27307 (11) were amplified in a PCR reaction and either Sanger sequenced and/or *de novo* Pyrosequenced.

Bones were ground and DNA extracted following the protocol described below for chickens, except that due to the small size of the rat bones, the bone surface was not abraded prior to pulverizing. Primers used in the Pyrosequencing PCR reaction were: *LinF* 5’-TCTTCTAGGAGTATGCCTTATAGT-biotinylated-3’ and *LinR* 5’-TGAGAATGCTGTTAAAGTGTC-3’. The sequencing primer was *LinS* 5’-ATAGTACAAATTATCACAGG-3’. Overlapping primers used in the Sanger sequencing reaction were: *RrF2* 5’ – TTAATCACTCCTTCATTGACCTTCC -3’ and *RrR2* 5’- AGCCGTAGTTTACGTCTCGGCAG -3’; *RrF3* 5’ – TTAACAGCATTCTCATCAGTTAC -3’ and *RrR3* 5’- GTTGCTATGACTGCAAATA -3’.

PCR reactions were 25 µl for Sanger sequencing and 50 µl for Pyrosequencing. PCR reactions contained variable concentrations of reagents on a sample-by-sample basis: ddH_2_0, 1 x buffer PCR Gold Buffer, 2.5-4 mM MgCl_2_, 0.2-0.3 mM dNTPs, 0.2-0.3 µM each primer, 1-2 U *Taq* DNA polymerase (AmpliTaq Gold), 0.5 µg/µl BSA, and in some cases where inhibition was present 0.5% Tween 20 and 0.7 M betaine. PCR cycling was as follows: 5min initial denaturation at 95°C, followed by 50 cycles of 35s at 95°C, 35s at 58°C, 45s at 72°C, and a 5min final extension at 72°C. Multiple PCRs from the same extracts, and different extracts from the same individual (where available), were sequenced.

Pyrosequencing products were *de novo* sequenced on a PyroMark Q24 following the manufacturer’s guidelines. Inspection of pyrograms and interpretation of results was conducted in PyroMark Q24 Analysis software. The passed peak height threshold was set to 20 and the checked peak height threshold was set to 10.

Sanger sequenced products were cleaned of unincorporated dNTPs with FastAP Thermosensitive Alkaline Phosphatase and Exonuclease I (ThermoScientific, Pittsburgh, USA) following the manufacturer’s protocol. Products were sequenced in both directions on an Applied Biosystems ABI 3730 using 3µl of template. Chromatograms were inspected and concatenated in Geneious v6.1.6.

The ancient DNA sequences were aligned with the *Rattus* species dataset and their identifications were determined by comparison of SNPs between target and known sequences. Where sequences deviated from expected variation (on visual inspection), we performed a sequence search in the GenBank nucleotide database using a BLASTN algorithm (www.ncbi.nlm.nih.gov/BLAST) to search for highly similar sequences.

**D) Preparation of rodent bone for ZooMS collagen fingerprinting**

For both modern reference specimens of murid rodents (see Methods section of main text), and for archaeological specimens, established methods were used to obtain collagen fingerprints (12). Bone was decalcified using 0.6 M hydrochloric acid for 18 hours and exchanged into 50 mM ammonium bicarbonate on 10 kDa molecular weight cut-off ultrafilters. This sample was digested with the protease trypsin overnight at 37^o^C. Following digestion into peptides, the samples were cleaned with C18 ziptips eluted into 50% acetonitrile/0.1% trifluoroacetic acid (TFA), dried by evaporation, and resuspended with 10 µL 0.1% TFA. 1 µL of samples were co-crystallized with α-cyano-hydroxycinnamic acid solution (10 mg/mL in 50% ACN/0.1% TFA) on a stainless steel MALDI target plate. MALDI analysis was carried out using a Bruker Ultraflex II with up to 2000 laser acquisitions.

**References**

1. Miao Y-W, Peng M-S, Wu G-S, Ouyang Y-N, Yang Z-Y, Yu N, et al. Chicken domestication: an updated perspective based on mitochondrial genomes. Heredity. 2013;110(3):277–82.

2. Thomson VA, Lebrasseur O, Austin JJ, Hunt TL, Burney DA, Denham T, et al. Using ancient DNA to study the origins and dispersal of ancestral Polynesian chickens across the Pacific. Proceedings of the National Academy of Sciences of the United States of America. 2014;111:4826-31.

3. Katoh K, Misawa K, Kuma K, Miyata T. MAFFT: a novel method for rapid multiple sequence alignment based on fast Fourier transform. Nucleic Acids Research. 2002;30(14):3059–66.

4. Dabney J, Knapp M, Glocke I, Gansauge M-T, Weihmann A, Nickel B, et al. Complete mitochondrial genome sequence of a Middle Pleistocene cave bear reconstructed from ultrashort DNA fragment. Proceedings of the National Academy of Sciences of the United States of America. 2013;110(39):15758–63.

5. Meyer M, Kircher M. Illumina sequencing library preparation for highly multiplexed target capture and sequencing. Cold Spring Harbor Protocols. 2010;2010(6):1–10.

6. Kircher M, Sawyer S, Meyer M. Double indexing overcomes inaccuracies in multiplex sequencing on the Illumina platform. Nucleic Acids Research. 2012;40(1):e3.

7. Lindgreen S. AdapterRemoval: easy cleaning of next-generation sequencing reads. BMC Research Notes. 2012;5(1):337.

8. Li H, Durbin R. Fast and accurate short read alignment with Burrows-Wheeler transform. Bioinformatics. 2009;25(14):1754–60.

9. Schubert M, Ginolhac A, Lindgreen S, Thompson JF, Al-Rasheid KAS, Willerslev E, et al. Improving ancient DNA read mapping against modern reference genomes", BMC Genomics. 2012;13(1):178.

10. Aplin KP, Suzuki H, Chinen AA, Chesser T, ten Have J, Donnellan SC, et al. Multiple geographic origins of commensalism and complex dispersal history of black rats. PLoS ONE. 2011;6(11):e26357.

11. Robins JH, McLenachan PA, Phillips MJ, Craig L, Ross HA, Matisoo-Smith E. Dating of divergences within the Rattus genus phylogeny using whole mitochondrial genomes. Molecular Phylogenetics and Evolution. 2008;49(2):460–6.

12. Buckley M, Collins MJ, Thomas-Oates J, Wilson J. Species identification by analysis of bone collagen using matrix-assisted laser desorption/ionisation time-of-flight mass spectrometry. Rapid Communications in Mass Spectrometry. 2009;23(23):3848–54.
