# Supplementary material for: Reconstructing Asian faunal introductions to eastern Africa from multi-proxy biomolecular and archaeological datasets
Source: PLoS One. 2017 Aug 17;12(8):e0182565. doi: 10.1371/journal.pone.0182565 (PMC5560628; doi:10.1371/journal.pone.0182565)
Supplement: S1 Table — (DOCX) [file pone.0182565.s002.docx]

**S1 Table. Sites excavated by the Sealinks Project.**

|  | | | | | | | | **Faunal data^4^** | | | |
| --- | --- | --- | --- | --- | --- | --- | --- | --- | --- | --- | --- |
| **Site code/name (reference, if published)** | **Type^1^** | **Area** | **South** | **East** | **SL seasons** | **General chronology^2^** | **Analyst^3^** | | **Total NISP** | **Subset NISP** |  |
| MM/Mulungu wa Mawe | C | S Kenya hinterland | 3°40'13" | 39°44'29" | 2011 | aceramic LSA, MIA | N/A | | N/A | N/A |  |
| PYS/Panga ya Saidi (1, 2) | C | S Kenya hinterland | 3°40'70" | 39°44'32" | 2010, 2011, 2013 | aceramic LSA, MIA | MEP, NMK | | 5256 | 2259 |  |
| SC/Panga ya Mwandzumari (1) ^5^ | C | S Kenya hinterland | 3°41'48" | 39°44'18" | 2010 | aceramic LSA, MIA | N/A | | N/A | N/A |  |
| KK/Kwa Kipoko | O | S Kenya hinterland | 3°45'19" | 39°41'27" | 2011, 2013 | EIA | MEP | | 21 | 2 |  |
| PMZ/Panga ya Mizigo (2) | C | S Kenya hinterland | 3°50'83" | 39°40'66" | 2011, 2013 | aceramic LSA, MIA | MEP | | 968 | 566 |  |
| PLW/Pango la Watoro | C | Pemba (Zanzibar) | 4°53'3" | 39°40'46" | 2012 | LIA | MEP | | 184 | 89 |  |
| PK/Makangale Cave^6^ | C | Pemba (Zanzibar) | 4°54'5" | 39°41'19" | 2012 | MIA | RH, MEP | | 5163 | 1330 |  |
| RM/Ras Mkumbuu | O, U | Pemba (Zanzibar) | 5°11'46" | 39°39'46" | 2012 | LIA | MEP | | 57 | 11 |  |
| FK/Fukuchani (2, 3) | O | Unguja (Zanzibar) | 5°49'18" | 39°17'27" | 2011 | MIA | MEP | | 505 | 273 |  |
| UU/Unguja Ukuu (2, 3) | O, U | Unguja (Zanzibar) | 6°18'0" | 39°29'0" | 2011, 2012 | MIA | MEP | | 1571 | 758 |  |
| KC/Kuumbi Cave (4, 5) | C | Unguja (Zanzibar) | 6°21'40" | 39°32'33" | 2012 | aceramic LSA, MIA | MEP | | 6667 | 1874 |  |
| JS/Juani School (6) | O | Juani (Mafia) | 7°59'23" | 39°46'57" | 2012 | EIA | MEP | | 96 | 81 |  |
| PU/Ukunju Cave (7) | C | Juani (Mafia) | 7°59'57" | 39°46'33" | 2012 | MIA | MEP | | 23 | 10 |  |
| NMW/Nyamawi | O | Ngazidja (Comoros) | 11°22'26" | 43°22'29" | 2013 | MIA | MEP | | 3 | 3 |  |
| SMA/Old Sima | O, U | Anjouan (Comoros) | 12°12'34" | 44°16'7" | 2013 | MIA | MEP | | 122 | 68 |  |
| MHLK/Mahilaka | O, U | NW Madagascar | 13°48'18" | 48°18'45" | 2013 | MIA | N/A | | N/A | N/A |  |

^1^ C = Cave, O = open-air, U = urban sites, or larger open-air sites that served as ports

^2^ LSA=Later Stone Age (up to c. 600 CE), EIA=Early Iron Age (CE 100-600); MIA=Middle Iron Age (CE 600-1000); LIA=Later Iron Age (CE 1000-1650).

^3^ MEP = Prendergast, RH = Hutterer, NMK = Mwebi + National Museums of Kenya staff; N/A = not available since no formal analysis conducted, specimens selected by excavators

^4^ Total NISP (Number of Identified Specimens) excludes fish and mollusk. Subset NISP excludes human remains and those not identified to order or lower taxonomic levels (e.g., "Mammal Size 3").

^5^ Previously published as Sinseme Cave.

^6^ Previously published as Mapangani Cave, Pango la Kijiji, or Pango la Kijiji Mapangani.

**References**

1. Helm R, Crowther A, Shipton C, Tengeza A, Fuller DQ, Boivin N. Exploring agriculture, interaction and trade on the eastern African littoral: preliminary results from Kenya. Azania: Archaeological Research in Africa. 2012;47(1):39–63.

2. Crowther A, Prendergast ME, Fuller DQ, Boivin N. Subsistence mosaics, forager-farmer interactions, and the transition to food production in eastern Africa. Quaternary International. 2017.

3. Prendergast ME, Quintana Morales EM, Crowther A, Horton MC, Boivin NL. Dietary Diversity on the Swahili Coast: The Fauna from Two Zanzibar Trading Locales. International Journal of Osteoarchaeology. 2017.

4. Shipton C, Crowther A, Kourampas N, Prendergast M, Horton MC, Douka K, et al. Reinvestigation of Kuumbi Cave, Zanzibar, reveals Later Stone Age coastal habitation, early Holocene abandonment and Iron Age reoccupation. Azania: Archaeological Research in Africa. 2016;51(2):197-233.

5. Prendergast ME, Rouby H, Punnwong P, Marchant R, Crowther A, Kourampas N, et al. Continental Island Formation and the Archaeology of Defaunation on Zanzibar, Eastern Africa. PLOS ONE. 2016;11(2):e0149565.

6. Crowther A, Faulkner P, Prendergast M, Quintana Morales E, Horton MC, Wilmsen E, et al. Coastal subsistence, island colonization and maritime population dispersal in eastern African prehistory. Journal of Island and Coastal Archaeology. 2016;11(2):211-37.

7. Crowther A, Horton MC, Kotarba-Morley A, Prendergast M, Quintana Morales E, Wood M, et al. Iron Age agriculture, fishing and trade in the Mafia Archipelago, Tanzania: new evidence from Ukunju Cave. Azania: Archaeological Research in Africa. 2014;49:21–44.
