# Supplementary material for: Reconstructing Asian faunal introductions to eastern Africa from multi-proxy biomolecular and archaeological datasets
Source: PLoS One. 2017 Aug 17;12(8):e0182565. doi: 10.1371/journal.pone.0182565 (PMC5560628; doi:10.1371/journal.pone.0182565)
Supplement: S2 Table — (DOCX) [file pone.0182565.s003.docx]

**S2 Table. Previously excavated sites included in the present analysis.**

| **Site code/name (reference)** | **Type^1^** | **Area** | **South** | **East** | **General chronology** | **Site PI^2^** | **Analyst^3^** | **NISP^4^** |
| --- | --- | --- | --- | --- | --- | --- | --- | --- |
| MTSE/Mtsengo (1) | O | S Kenya hinterland | 3°43'28" | 39°37’15" | MIA-LIA | RMH | NM | 1825 |
| MBYN/Mbuyuni (1) | O | S Kenya hinterland | 3°56'53" | 39°31'58" | LIA | RMH | NM | 990 |
| CHO/Chombo (1) | O | S Kenya hinterland | 4°7'0" | 39°28'60" | MIA | RMH | NM | 718 |
| VMB/Vumba Kuu (2) | O,U | S Kenya coast | 5°13'48" | 39°49'48" | 15th C CE | SWJ | EQM | 548 |
| UU/Unguja Ukuu (3) | O,U | Unguja (Zanzibar) | 6°18'0" | 39°29'0" | MIA | MCH | NM | 254 |
| SM/Songo Mnara (4, 5) | O,U | Pemba (Zanzibar) | 9°4'12" | 39°34'12" | 15th C CE | SWJ/ JBF | EQM | 304 |
| DMB/Dembeni (6) | O, U | Mayotte (Comoros) | 12°50'33" | 45°11'5" | MIA | CA | WVN | 417 |

^1^ C = Cave, O = open-air, U = urban sites, or larger open-air sites that served as ports

^2^ RMH = Helm; SWJ = Wynne-Jones; JBF = Fleisher; MCH = Horton; CA = Claude Allibert

^3^ NM = Nina Mudida; EQM = Quintana Morales; WVN = Van Neer

^4^ NISP excludes fish, molluscs, humans, and animals only referred to by size class

**References**

1. Helm R. Conflicting histories: the archaeology of the iron-working, farming communities in the central and southern coast region of Kenya [Unpublished PhD Thesis]. Bristol: University of Bristol; 2000.

2. Wynne-Jones S. Remembering and reworking the Swahili Diwanate: the role of objects and places at Vumba Kuu. International Journal of African Historical Studies. 2010;43(3):407–27.

3. Horton MC. Zanzibar and Pemba: The archaeology of an Indian Ocean trading village London: BIEA; in press.

4. Wynne-Jones S. The public life of the Swahili stonehouse, 14th–15th centuries AD. Journal of Anthrolopogical Archaeology. 2013;32:759–73.

5. Fleisher J. The complexity of public space at the Swahili town of Songo Mnara, Tanzania. Journal of Anthrolopogical Archaeology. 2014;35:1–22.

6. Allibert C, Argant A, Argant J. Le site de Dembeni (Mayotte, Archipel des Comores), mission 1984. Études Océan Indien. 1989;11:63–172.
