# Supplementary material for: Reconstructing Asian faunal introductions to eastern Africa from multi-proxy biomolecular and archaeological datasets
Source: PLoS One. 2017 Aug 17;12(8):e0182565. doi: 10.1371/journal.pone.0182565 (PMC5560628; doi:10.1371/journal.pone.0182565)
Supplement: S3 Table — Incorrect genus identifications resulting from 500 test "libraries" obtained from whole mtDNA genomes of the genus Gallus. See text for explanation of experimental method. (DOCX) [file pone.0182565.s004.docx]

**S3 Table. Results of experimental study of false positives.**

Incorrect genus identifications resulting from 500 test "libraries" obtained from whole mtDNA genomes of the genus *Gallus*. See text for explanation of experimental method.

|  | **Number of reads per library** | | | | |
| --- | --- | --- | --- | --- | --- |
| **p-value** | 50 | 100 | 200 | 500 | 1000 |
| 0.9 | 0 | 0 | 0 | 0 | 0 |
| 0.8 | 0 | 0 | 0 | 0 | 0 |
| 0.5 | 0 | 0 | 0 | 0 | 0 |
| 0.25 | 27 | 12 | 3 | 0 | 0 |
