# Supplementary material for: Reconstructing Asian faunal introductions to eastern Africa from multi-proxy biomolecular and archaeological datasets
Source: PLoS One. 2017 Aug 17;12(8):e0182565. doi: 10.1371/journal.pone.0182565 (PMC5560628; doi:10.1371/journal.pone.0182565)
Supplement: S4 Table — (DOCX) [file pone.0182565.s005.docx]

**S4 Table. Reference specimens for analysis of tooth morphology.**

| **Taxon** | **Common name(s)** | **Number of studied specimens (CERoPATH, Montpellier)** |
| --- | --- | --- |
| *Rattus argentiventer* | Ricefield rat | 34 |
| *Rattus exulans* | Polynesian rat, Pacific rat | 29 |
| *Rattus losea* | Lesser ricefield rat | 29 |
| *Rattus nitidus* | Himalayan field rat | 13 |
| *Rattus norvegicus* | Brown rat | 28 |
| *Rattus rattus* lineage I | Black rat | 29 |
| *Rattus rattus* lineage II | Tanezumi rat, Asian rat | 22 |
| *Rattus rattus* clade 3 | Tanezumi rat, Asian rat | 18 |
| *Bandicota indica* | Greater bandicoot rat | 28 |
| *Bandicota savilei* | Savile’s bandicoot rat | 28 |
| *Berylmys berdmorei* | Small white-toothed rat | 25 |
| *Berylmys bowersi* | Bower’s white-toothed rat | 28 |
| *Leopoldamys edwardsi* | Edward’s long-tailed giant rat | 24 |
| *Maxomys surifer* | Red spiny rat | 28 |
| *Niviventer fulvescens* | Chestnut white-bellied rat | 34 |
