# Supplementary material for: Reconstructing Asian faunal introductions to eastern Africa from multi-proxy biomolecular and archaeological datasets
Source: PLoS One. 2017 Aug 17;12(8):e0182565. doi: 10.1371/journal.pone.0182565 (PMC5560628; doi:10.1371/journal.pone.0182565)
Supplement: S5 Table — (DOCX) [file pone.0182565.s006.docx]

**S5 Table. Reference specimens for ZooMS collagen fingerprinting.**

| **Institution** | **Catalog number** | **Origin** | **Taxon** | **Common name** |
| --- | --- | --- | --- | --- |
| Manchester | C1748 | U.K. | *Rattus rattus* | Black rat |
| Manchester | C2008 | U.K. | *Rattus norvegicus* | Brown rat |
| ZMFK | 2001.285 | South Africa | *Mastomys coucha* | Southern multimammate mouse |
| ZMFK | 2001.285 | South Africa | *Mastomys coucha* | Southern multimammate mouse |
| ZMFK | 2001.252 | South Africa | *Mastomys coucha* | Southern multimammate mouse |
| ZMFK | 2001.183 | South Africa | *Mus/Nannomys minutoides* | African pygmy mouse |
| ZMFK | 2001.189 | South Africa | *Mus/Nannomys minutoides* | African pygmy mouse |
| MNHN | ZM-MO-1991-735 | Tanzania | *Thallomys paedulcus* | Acacia rat |
| RBINS | 18281 | DR Congo | *Aethomys kaiseri* | Kaiser’s rock rat |
| RBINS | 13385 | DR Congo | *Gerbilliscus validus* (formerly *Tatera valida)* | Savanna gerbil |
| RBINS | 18484 | DR Congo | *Otomys tropicalis* | Tropical vlei rat |
| RBINS | 567B | Africa | *Thallomys paeduculus* | Acacia rat |
| RBINS | 196450 | Java | *Rattus exulans browni* | Pacific rat |
| RBINS | 21027 | Andaman Is. | *Rattus rattus tanezumi* | Black rat, tanezumi (Asian) variety |

Manchester, University of Manchester Paleoproteomics Laboratory collections; MNHN, Museum National d'Histoire Naturelle. Paris, France; RBINS, Royal Belgian Institute for Natural Sciences, Brussels, Belgium; ZMFK, Zoological Research Museum Alexander Koenig (ZMFK) Bonn, Germany.
