# Supplementary material for: Reconstructing Asian faunal introductions to eastern Africa from multi-proxy biomolecular and archaeological datasets
Source: PLoS One. 2017 Aug 17;12(8):e0182565. doi: 10.1371/journal.pone.0182565 (PMC5560628; doi:10.1371/journal.pone.0182565)
Supplement: S6 Table — Results of multiple ancient DNA analyses, with radiocarbon dates where available. Sites ordered from north to south. (DOCX) [file pone.0182565.s007.docx]

**S6 Table. Detailed results for bird specimens.**

Sites ordered from north to south.

| **Site** | **Context** | **Original Attribution** | **Element** | **DNA μg/mL** | **GG144F/ 387R** | **12S** | **BLAST and *p* value** | **uncal BP date (bold = direct)** | **cal CE range** | **Specimen ID** |
| --- | --- | --- | --- | --- | --- | --- | --- | --- | --- | --- |
| MM | 101 | Aff. Galliformes | pelvis | 23.8 | Gallus | Human (fail) |  | **133.3±0.4 (Wk-41392)** | **1695-1930** | OL533chi |
| MM | 101 | Aff. Galliformes | tibiotarsus | 7.87 | Gallus | not tested |  | assoc. with 133.3±0.4 (Wk-41392) | 17th-20th C | OL536chi |
| MM | 101 | *G. gallus* | coracoid | 37.4 | Non-match | Human (fail) |  | assoc. with 133.3±0.4 (Wk-41392) | 17th-20th C | OL526chi |
| MM | 101 | Bird | humerus | 6.14 | Non-match | *Bycanistes brevis* |  | assoc. with 133.3±0.4 (Wk-41392) | 17th-20th C | OL528chi |
| MM | 102 | Bird | vertebral row | 51 | Gallus | not tested |  | **133.6±0.5 (Wk-41393)** | **1695-1930** | OL524chi |
| MM | 102 | Aff. Galliformes | femur | 8.54 | Gallus | Human (fail) |  | assoc. with 133.6±0.5 (Wk-41393) | 17th-20th C | OL534chi |

| **Site** | **Context** | **Original Attribution** | **Element** | **DNA μg/mL** | **GG144F/ 387R** | **12S** | **BLAST and *p* value** | **uncal BP date (bold = direct)** | **cal CE range** | **Specimen ID** |
| --- | --- | --- | --- | --- | --- | --- | --- | --- | --- | --- |
| MM | 102 | Bird | vertebra | 21 | Non-match | Human (fail) |  | assoc. with 133.6±0.5 (Wk-41393) | 17th-20th C | OL529chi |
| MM | 102 | *G. gallus* | carpometacarpus | 15.8 | Non-match | Human (fail) |  | assoc. with 133.6±0.5 (Wk-41393) | 17th-20th C | OL525chi |
| PYS | 307 | Galliformes | ulna | 2.29 | Non-match | Unreadable (fail) | fail | overlies 1213±23 (OxA-29285), underlies 388±27 (OxA-30803) | 8th-17th C^1^ | RA42/JK3005 |
| PMZ | 301 | *G. gallus* | scapula | 8.79 | Gallus | not tested |  | none | likely modern | OL521chi |
| FK | 12/003 | Galliformes | coracoid | 1.24 | Non-match | Human (fail) | *Gallus* 0.5352, *Bambusicola* 0.4648 | overlies 1325±23 (OxA-31426) | 7th-8th C or later | OL531chi/JK3000 |

| **Site** | **Context** | **Original Attribution** | **Element** | **DNA μg/mL** | **GG144F/ 387R** | **12S** | **BLAST and *p* value** | **uncal BP date (bold = direct)** | **cal CE range** | **Specimen ID** |
| --- | --- | --- | --- | --- | --- | --- | --- | --- | --- | --- |
| UU | 10/004 | Galliformes | tibiotarsus | 24.6 | Non-match | fail | fail | overlies 1282±24 (OxA-29310) | 7th-9th C or later | RA47/JK2989 |
| UU | 11/004 | Galliformes | coracoid | 5.49 | Non-match | Human (fail) | fail | assoc. with 1178±25 (OxA-27517) | 8th-10th C or later | OL523chi/JK2997 |
| UU | 11/015 | Galliformes | tarsometatarsus | 1.84 | Non-match | Human (fail) | fail | underlies 1280±26 (OxA-27515) | 7th-9th C | OL530chi/JK2988 |
| UU | 13/002 | Galliformes | tarsometatarsus | 6.7 | Non-match | Human (fail) | *Syrmaticus* 0.9920, *Gallus* 0.0026 | none | 8th-10th C | OL522chi/JK2992 |
| UU | 13/003 | Galliformes | coracoid | 13.4 | Non-match | Human (fail) | fail | none | 8th-10th C | OL532chi/JK2999 |
| UU | 1412 | *G. gallus* | limb bone | 3.36 | Non-match | Human (fail) | *Gallus* 1, *Bambusicola* 1.13 E-66 | overlies 1244±27 (OxA-27518), underlies 1151±26 (OxA-27520) | 8th-10th C | RA46/OL511chi/JK2995 |

| **Site** | **Context** | **Original Attribution** | **Element** | **DNA μg/mL** | **GG144F/ 387R** | **12S** | **BLAST and *p* value** | **uncal BP date (bold = direct)** | **cal CE range** | **Specimen ID** |
| --- | --- | --- | --- | --- | --- | --- | --- | --- | --- | --- |
| KC | 1002 | Cf. G. gallus | coracoid | out of range | Non-match | Unreadable (fail) | *Numida* 1, *Acryllium* 7.24 E-55 | overlies 590±50 (X6696P) | 14th-15th C or later | RA44/JK3001 |
| KC | 1003 | Cf. Galliformes | limb bone | 11.2 | Non-match | Human (fail) | *Syrmaticus* 0.9925, *Arborophila* 0.003 | assoc. with 590±50 (X6696P) | 14th-15th C | RA49/OL519chi/JK2994 |
| KC | 1007 | Aff. Galliformes | scapula | 8.11 | Non-match | Human (fail) | *Polyplectron* 0.9976, *Numida* 0.0010 | assoc. with 685±85 (X6698P), 1360±125 (X6697P) | 6th-8th C or later | RA48/OL509chi/JK2993 |
| KC | 1015 | Galliformes | tibiotarsus | 0.92 | Non-match | Human (fail) | fail | assoc. with 5082±23 (Wk-40962), overlies 1899±20 (Wk-40961)^2^ | indeterminate^2^ | RA50/OL512chi/JK2991 |
| KC | 1017 | Galliformes | tibiotarsus | 2.71 | Non-match | Unreadable (fail) | fail | overlies 14221±62 (Wk-40632), underlies 1899±20 (Wk-40961)^2^ | indeterminate^2^ | RA43/JK3006 |
| KC | 1018 | Galliformes | carpometacarpus | 0.603 | Non-match | fail | fail | overlies 14221±62 (Wk-40632), underlies 1899±20 (Wk-40961)^2^ | indeterminate^2^ | RA45/OL510chi/JK2990 |

| **Site** | **Context** | **Original Attribution** | **Element** | **DNA μg/mL** | **GG144F/ 387R** | **12S** | **BLAST and *p* value** | **uncal BP date (bold = direct)** | **cal CE range** | **Specimen ID** |
| --- | --- | --- | --- | --- | --- | --- | --- | --- | --- | --- |
| SMA | 8 | *G. gallus* | coracoid |  | not tested | not tested | fail | assoc. with 1230±24 (OxA-30711) | 8th-9th C | JK2998 |
| SMA | 12/13 | *G. gallus* | coracoid |  | not tested | not tested | *Gallus* 0.4996, *Arborophila* 0.4591 | assoc. with 1163±23 (OxA-30709), 1276±24 (OxA-30708) | 7th-10th C | JK2996 |
| MHLK | 2005 | Cf. *G. gallus* | indeterminate |  | Non-match | Human (fail) | fail | underlies 879±24 (OxA-30386), overlies 1023±26 (OxA-30557) | 10th-13th C | OL518chi/JK3004 |
| MHLK | 2006 | Cf. *G. gallus* | limb bone |  | Non-match | Human (fail) | *Gallus* (0.9984), *Bambusicola* (0.001) | underlies 879±24 (OxA-30386), overlies 1023±26 (OxA-30557) | 10th-13th C | OL514chi/JK3007 |
| MHLK | 2007 | Cf. *G. gallus* | femur |  | Non-match | Human (fail) | fail | underlies 879±24 (OxA-30386), overlies 1023±26 (OxA-30557) | 10th-13th C | OL513chi/JK3008 |
| MHLK | 2007 | Cf. *G. gallus* | tibiotarsus |  | Non-match | Human (fail) | fail | underlies 879±24 (OxA-30386), overlies 1023±26 (OxA-30557) | 10th-13th C | OL517chi/JK3003 |
| MHLK | 2011 | Cf. *G. gallus* | tarsometatarsus |  | Non-match | Human (fail) | fail | underlies 879±24 (OxA-30386), overlies 1023±26 (OxA-30557) | 10th-13th C | OL515chi/JK3002 |
