# Supplementary material for: Reconstructing Asian faunal introductions to eastern Africa from multi-proxy biomolecular and archaeological datasets
Source: PLoS One. 2017 Aug 17;12(8):e0182565. doi: 10.1371/journal.pone.0182565 (PMC5560628; doi:10.1371/journal.pone.0182565)
Supplement: S7 Table — (DOCX) [file pone.0182565.s008.docx]

**Table S7: Total reads used in the BLAST analysis and results of Burrows-Wheeler Alignments (BWA)**

| **Library** | **Total reads** | **NreadsG** | **MeanLen** | **NreadsC** | **GC_overlap** | **MAPQD_GC** | **NreadsH** | **GN_overlap** | **MAPQD_HC** | **NreadsMTdb** |
| --- | --- | --- | --- | --- | --- | --- | --- | --- | --- | --- |
| JK1996 | 23491782 | 10622 | 26.86 | 10264 | 0.633199511 | -2.112724084 | 12201 | 0.304785459 | -9.905909352 | 310 |
| JK2988 | 20140326 | 19066 | 21.96 | 20210 | 0.696071566 | -3.781296388 | 25292 | 0.358770444 | -9.347659339 | 585 |
| JK2989 | 18755250 | 11708 | 22.23 | 12516 | 0.732818294 | -2.64892937 | 29574 | 0.385512898 | -19.4638872 | 529 |
| JK2990 | 24084682 | 19942 | 23.4 | 19375 | 0.680501321 | -2.564593548 | 131953 | 0.364883968 | -26.9584928 | 694 |
| JK2991 | 18993388 | 4727 | 20.06 | 5461 | 0.688312381 | -3.233473723 | 6289 | 0.35658692 | -9.024010176 | 165 |
| JK2992 | 21594246 | 13391 | 30.63 | 11189 | 0.57108722 | -0.11082313 | 17622 | 0.250732592 | -15.61905573 | 346 |
| JK2993 | 19541886 | 34929 | 48.78 | 29574 | 0.633725893 | 7.129607087 | 14124 | 0.114702045 | -8.869300481 | 482 |
| JK2994 | 19558892 | 21921 | 45.06 | 19242 | 0.639857538 | 5.712763746 | 16210 | 0.141127589 | -13.91776681 | 309 |
| JK2995 | 21112582 | 73822 | 40.77 | 31288 | 0.360455167 | 10.22938507 | 29784 | 0.067712994 | -17.35505641 | 833 |
| JK2997 | 22762292 | 9697 | 22.95 | 10847 | 0.721796994 | -3.11809717 | 34101 | 0.376595528 | -22.79733732 | 388 |
| JK2998 | 21933284 | 11509 | 20.51 | 12417 | 0.679322936 | -3.167753886 | 14902 | 0.352544728 | -9.049993289 | 362 |
| JK2999 | 21164834 | 8939 | 25.16 | 9674 | 0.69271334 | -0.852077734 | 10226 | 0.300399201 | -9.235869353 | 302 |
| JK3000 | 20999570 | 14494 | 32.32 | 11842 | 0.553959435 | -0.477790914 | 12441 | 0.251763061 | -7.7070975 | 416 |
| JK3001 | 22557020 | 275075 | 45.52 | 210158 | 0.592943521 | 11.29313659 | 21939 | 0.023588497 | -7.896895939 | 1742 |
| JK3002 | 18515098 | 9840 | 22.83 | 9934 | 0.674039505 | -1.828870546 | 11157 | 0.332796902 | -7.643183652 | 338 |
| JK3003 | 20392820 | 20019 | 20.41 | 21053 | 0.755927971 | -1.427112526 | 24807 | 0.391946946 | -6.709235296 | 937 |
| JK3004 | 18057696 | 15440 | 20.26 | 16399 | 0.762915859 | -1.303250198 | 19103 | 0.394496413 | -6.219337277 | 679 |
| JK3005 | 20448628 | 23360 | 20.14 | 23907 | 0.744447804 | -2.422721379 | 28691 | 0.38362542 | -7.193161619 | 997 |
| JK3006 | 23776088 | 16304 | 21.12 | 17043 | 0.691283715 | -2.799448454 | 24998 | 0.362526686 | -11.71689735 | 554 |
| JK3007 | 17610584 | 47324 | 21.96 | 43790 | 0.704407167 | 0.017880795 | 50209 | 0.342611463 | -6.207871099 | 1904 |
| JK3008 | 20180636 | 10117 | 20.96 | 11148 | 0.736244755 | -2.102529602 | 12915 | 0.38243195 | -7.329461866 | 380 |

**NreadsT** = total number of reads; **NreadsG** = total number of reads mapping the Galgal4 assembly; **MeanLen** = mean read length; **NreadsC** = total number for reads mapping the *Coturnix japonica* assembly; **GC_overlap** = proportion of reads that map to Galgal4 also mapping the *Coturnix* assembly; **MAPQD_GC** = difference in mapping quality for each read mapping to both Galgal4 and *Coturnix*; **NreadsH** = number of reads mapping to Hg19; **NreadsMTdb** = number of reads mapping to the 957 mtDNA genomes of birds.
