# Supplementary material for: Reconstructing Asian faunal introductions to eastern Africa from multi-proxy biomolecular and archaeological datasets
Source: PLoS One. 2017 Aug 17;12(8):e0182565. doi: 10.1371/journal.pone.0182565 (PMC5560628; doi:10.1371/journal.pone.0182565)
Supplement: S8 Table — Results of ancient DNA analysis, ZooMS collagen fingerprinting, and tooth morphology, with radiocarbon dates where available. Sites ordered from north to south. (DOCX) [file pone.0182565.s009.docx]

**S8 Table. Detailed results for rodent specimens.**

Sites ordered from north to south.

| **Site** | **Context** | **Original Attribution^1^** | **Element** | **Dental analysis** | **ZooMS** | **aDNA** | **uncal BP date (bold = direct)** | **cal CE range** | **Specimen ID** |
| --- | --- | --- | --- | --- | --- | --- | --- | --- | --- |
| PYS | 205 | Rat | vertebra |  |  | Fail | none | unknown (LSA-MIA interface) | RAT_01 |
| PYS | 301 | Cf. *Rattus*/Muridae | pelvis |  |  | Not *Rattus* | none | likely modern | RAT_62 |
| PYS | 301 | Cf. *Rattus*/Muridae | pelvis |  | *Group 1 (Mastomys sp.)* |  | none | likely modern | NB17 |
| PYS | 301 | Cf. *Rattus*/Muridae | femur |  | *Group 1 (Mastomys sp.)* |  | none | likely modern | NB18 |
| PYS | 301 | Cf. *Rattus*/Muridae | tibia |  | *Group 1 (Mastomys sp.)* |  | none | likely modern | NB19 |
| PYS | 301 | Cf. *Rattus*/Muridae | femur |  | Group 3 (Unknown; Not *Rattus)* |  | none | likely modern | NB20 |
| PYS | 301 | Cf. *Rattus*/Muridae | pelvis |  | *Group 1 (Mastomys sp.)* |  | none | likely modern | NB21 |
| PYS | 301 | Cf. *Rattus*/Muridae | maxilla |  | *Group 2 (Gerbilliscus sp.)* |  | none | likely modern | NB89 |
| PYS | 302 | Cf. *Rattus*/Muridae | mandible | Not *Rattus* | *Group 1 (Mastomys sp.)* |  | overlies 1213±23 (OxA-29285), underlies 388±27 (OxA-30803) | 8th-17th C^2^ | NB156 |
| PYS | 302 | Cf. *Rattus*/Muridae | mandible |  | *Group 1 (Mastomys sp.)* |  | overlies 1213±23 (OxA-29285), underlies 388±27 (OxA-30803) | 8th-17th C^2^ | NB166 |
| **Site** | **Context** | **Original Attribution^1^** | **Element** | **Dental analysis** | **ZooMS** | **aDNA** | **uncal BP date (bold = direct)** | **cal CE range** | **Specimen ID** |
| PYS | 302 | Cf. *Rattus*/Muridae | incisor |  | *Group 1 (Mastomys sp.)* |  | overlies 1213±23 (OxA-29285), underlies 388±27 (OxA-30803) | 8th-17th C^2^ | NB174 |
| PYS | 302 | Cf. *Rattus*/Muridae | tibia |  | Fail |  | overlies 1213±23 (OxA-29285), underlies 388±27 (OxA-30803) | 8th-17th C^2^ | NB90 |
| PYS | 302 | Cf. *Rattus*/Muridae | vertebra |  | *Group 2 (Gerbilliscus sp.)* |  | overlies 1213±23 (OxA-29285), underlies 388±27 (OxA-30803) | 8th-17th C^2^ | NB91 |
| PYS | 302 | Cf. *Rattus*/Muridae | tibia |  | Fail |  | overlies 1213±23 (OxA-29285), underlies 388±27 (OxA-30803) | 8th-17th C^2^ | NB92 |
| PYS | 302 | Cf. *Rattus*/Muridae | vertebra |  | Fail |  | overlies 1213±23 (OxA-29285), underlies 388±27 (OxA-30803) | 8th-17th C^2^ | NB93 |
| PYS | 302 | Cf. *Rattus*/Muridae | femur |  | *Group 1 (Mastomys sp.)* |  | overlies 1213±23 (OxA-29285), underlies 388±27 (OxA-30803) | 8th-17th C^2^ | NB94 |
| PYS | 302 | Cf. *Rattus*/Muridae | femur |  | Fail |  | overlies 1213±23 (OxA-29285), underlies 388±27 (OxA-30803) | 8th-17th C^2^ | NB95 |
| PYS | 302 | Cf. *Rattus*/Muridae | vertebra |  | Fail |  | overlies 1213±23 (OxA-29285), underlies 388±27 (OxA-30803) | 8th-17th C^2^ | NB96 |
| PYS | 302 | Cf. *Rattus*/Muridae | vertebra |  | Fail |  | overlies 1213±23 (OxA-29285), underlies 388±27 (OxA-30803) | 8th-17th C^2^ | NB97 |
| PYS | 302 | Cf. *Rattus*/Muridae | radius |  | *Group 2 (Gerbilliscus sp.)* |  | overlies 1213±23 (OxA-29285), underlies 388±27 (OxA-30803) | 8th-17th C^2^ | NB98 |
| PYS | 304 | Cf. *Rattus*/Muridae | mandible | Not *Rattus* | *Group 1 (Mastomys sp.)* |  | overlies 1213±23 (OxA-29285), underlies 388±27 (OxA-30803) | 8th-17th C^2^ | NB164 |
| **Site** | **Context** | **Original Attribution^1^** | **Element** | **Dental analysis** | **ZooMS** | **aDNA** | **uncal BP date (bold = direct)** | **cal CE range** | **Specimen ID** |
| PYS | 304 | Cf. *Rattus*/Muridae | mandible |  | Fail |  | overlies 1213±23 (OxA-29285), underlies 388±27 (OxA-30803) | 8th-17th C^2^ | NB143 |
| PYS | 304 | Cf. *Rattus*/Muridae | mandible |  | Fail |  | overlies 1213±23 (OxA-29285), underlies 388±27 (OxA-30803) | 8th-17th C^2^ | NB144 |
| PYS | 304 | Cf. *Rattus*/Muridae | mandible |  | *Group 2 (Gerbilliscus sp.)* |  | overlies 1213±23 (OxA-29285), underlies 388±27 (OxA-30803) | 8th-17th C^2^ | NB145 |
| PYS | 304 | Cf. *Rattus*/Muridae | mandible |  | Fail |  | overlies 1213±23 (OxA-29285), underlies 388±27 (OxA-30803) | 8th-17th C^2^ | NB146 |
| PYS | 304 | Cf. *Rattus*/Muridae | cranium |  | *Group 1 (Mastomys sp.)* |  | overlies 1213±23 (OxA-29285), underlies 388±27 (OxA-30803) | 8th-17th C^2^ | NB147 |
| PYS | 304 | Cf. *Rattus*/Muridae | cranium |  | *Group 1 (Mastomys sp.)* |  | overlies 1213±23 (OxA-29285), underlies 388±27 (OxA-30803) | 8th-17th C^2^ | NB148 |
| PYS | 304 | Cf. *Rattus*/Muridae | mandible |  | *Group 1 (Mastomys sp.)* |  | overlies 1213±23 (OxA-29285), underlies 388±27 (OxA-30803) | 8th-17th C^2^ | NB128 |
| PYS | 305 | Cf. *Rattus*/Muridae | femur |  |  | Not *Rattus* | overlies 1213±23 (OxA-29285), underlies 388±27 (OxA-30803) | 8th-17th C^2^ | RAT_63 |
| PYS | 305 | Cf. *Rattus*/Muridae | tibia |  | *Group 1 (Mastomys sp.)* |  | overlies 1213±23 (OxA-29285), underlies 388±27 (OxA-30803) | 8th-17th C^2^ | NB22 |
| PYS | 305 | Cf. *Rattus*/Muridae | femur |  | Fail |  | overlies 1213±23 (OxA-29285), underlies 388±27 (OxA-30803) | 8th-17th C^2^ | NB23 |
| PYS | 305 | Cf. *Rattus*/Muridae | tibia |  | *Group 2 (Gerbilliscus sp.)* |  | overlies 1213±23 (OxA-29285), underlies 388±27 (OxA-30803) | 8th-17th C^2^ | NB24 |
| **Site** | **Context** | **Original Attribution^1^** | **Element** | **Dental analysis** | **ZooMS** | **aDNA** | **uncal BP date (bold = direct)** | **cal CE range** | **Specimen ID** |
| PYS | 305 | Cf. *Rattus*/Muridae | pelvis |  | Fail |  | overlies 1213±23 (OxA-29285), underlies 388±27 (OxA-30803) | 8th-17th C^2^ | NB25 |
| PYS | 305 | Cf. *Rattus*/Muridae | pelvis |  | *Group 1 (Mastomys sp.)* |  | overlies 1213±23 (OxA-29285), underlies 388±27 (OxA-30803) | 8th-17th C^2^ | NB26 |
| PYS | 305 | Cf. *Rattus*/Muridae | femur |  | Fail |  | overlies 1213±23 (OxA-29285), underlies 388±27 (OxA-30803) | 8th-17th C^2^ | NB27 |
| PYS | 305 | Cf. *Rattus*/Muridae | femur |  | *Group 2 (Gerbilliscus sp.)* |  | overlies 1213±23 (OxA-29285), underlies 388±27 (OxA-30803) | 8th-17th C^2^ | NB28 |
| PYS | 305 | Cf. *Rattus*/Muridae | femur |  | *Group 2 (Gerbilliscus sp.)* |  | overlies 1213±23 (OxA-29285), underlies 388±27 (OxA-30803) | 8th-17th C^2^ | NB29 |
| PYS | 305 | Cf. *Rattus*/Muridae | tibia |  | Fail |  | overlies 1213±23 (OxA-29285), underlies 388±27 (OxA-30803) | 8th-17th C^2^ | NB31 |
| PYS | 305 | Cf. *Rattus*/Muridae | femur |  | Fail |  | overlies 1213±23 (OxA-29285), underlies 388±27 (OxA-30803) | 8th-17th C^2^ | NB32 |
| PYS | 305 | Cf. *Rattus*/Muridae | tibia |  | *Group 2 (Gerbilliscus sp.)* |  | overlies 1213±23 (OxA-29285), underlies 388±27 (OxA-30803) | 8th-17th C^2^ | NB33 |
| PYS | 305 | Cf. *Rattus*/Muridae | tibia |  | *Group 2 (Gerbilliscus sp.)* |  | overlies 1213±23 (OxA-29285), underlies 388±27 (OxA-30803) | 8th-17th C^2^ | NB34 |
| PYS | 305 | Cf. *Rattus*/Muridae | humerus |  | *Group 1 (Mastomys sp.)* |  | overlies 1213±23 (OxA-29285), underlies 388±27 (OxA-30803) | 8th-17th C^2^ | NB35 |
| PYS | 305 | Cf. *Rattus*/Muridae | tibia |  | *Group 2 (Gerbilliscus sp.)* |  | overlies 1213±23 (OxA-29285), underlies 388±27 (OxA-30803) | 8th-17th C^2^ | NB36 |
| **Site** | **Context** | **Original Attribution^1^** | **Element** | **Dental analysis** | **ZooMS** | **aDNA** | **uncal BP date (bold = direct)** | **cal CE range** | **Specimen ID** |
| PYS | 305 | Cf. *Rattus*/Muridae | femur |  | *Group 1 (Mastomys sp.)* |  | overlies 1213±23 (OxA-29285), underlies 388±27 (OxA-30803) | 8th-17th C^2^ | NB37 |
| PYS | 305 | Cf. *Rattus*/Muridae | tibia |  | *Group 2 (Gerbilliscus sp.)* |  | overlies 1213±23 (OxA-29285), underlies 388±27 (OxA-30803) | 8th-17th C^2^ | NB38 |
| PYS | 305 | Cf. *Rattus*/Muridae | tibia |  | Fail |  | overlies 1213±23 (OxA-29285), underlies 388±27 (OxA-30803) | 8th-17th C^2^ | NB39 |
| PYS | 305 | Cf. *Rattus*/Muridae | femur |  | *Group 2 (Gerbilliscus sp.)* |  | overlies 1213±23 (OxA-29285), underlies 388±27 (OxA-30803) | 8th-17th C^2^ | NB40 |
| PYS | 305 | Cf. *Rattus*/Muridae | tibia |  | *Group 2 (Gerbilliscus sp.)* |  | overlies 1213±23 (OxA-29285), underlies 388±27 (OxA-30803) | 8th-17th C^2^ | NB41 |
| PYS | 305 | Cf. *Rattus*/Muridae | tibia |  | *Group 2 (Gerbilliscus sp.)* |  | overlies 1213±23 (OxA-29285), underlies 388±27 (OxA-30803) | 8th-17th C^2^ | NB42 |
| PYS | 305 | Cf. *Rattus*/Muridae | humerus |  | Fail |  | overlies 1213±23 (OxA-29285), underlies 388±27 (OxA-30803) | 8th-17th C^2^ | NB43 |
| PYS | 305 | Cf. *Rattus*/Muridae | femur |  | Fail |  | overlies 1213±23 (OxA-29285), underlies 388±27 (OxA-30803) | 8th-17th C^2^ | NB45 |
| PYS | 305 | Cf. *Rattus*/Muridae | femur |  | Fail |  | overlies 1213±23 (OxA-29285), underlies 388±27 (OxA-30803) | 8th-17th C^2^ | NB46 |

| **Site** | **Context** | **Original Attribution^1^** | **Element** | **Dental analysis** | **ZooMS** | **aDNA** | **uncal BP date (bold = direct)** | **cal CE range** | **Specimen ID** |
| --- | --- | --- | --- | --- | --- | --- | --- | --- | --- |
| PYS | 306 | Cf. *Rattus*/Muridae | humerus |  | Fail |  | overlies 1213±23 (OxA-29285), underlies 388±27 (OxA-30803) | 8th-17th C^2^ | NB10 |
| PYS | 306 | Cf. *Rattus*/Muridae | humerus |  | Fail |  | overlies 1213±23 (OxA-29285), underlies 388±27 (OxA-30803) | 8th-17th C^2^ | NB11 |
| PYS | 306 | Cf. *Rattus*/Muridae | humerus |  | Fail |  | overlies 1213±23 (OxA-29285), underlies 388±27 (OxA-30803) | 8th-17th C^2^ | NB12 |
| PYS | 306 | Cf. *Rattus*/Muridae | humerus |  | Fail |  | overlies 1213±23 (OxA-29285), underlies 388±27 (OxA-30803) | 8th-17th C^2^ | NB13 |
| PYS | 306 | Cf. *Rattus*/Muridae | humerus |  | Fail |  | overlies 1213±23 (OxA-29285), underlies 388±27 (OxA-30803) | 8th-17th C^2^ | NB14 |
| PYS | 306 | Cf. *Rattus*/Muridae | humerus |  | Fail |  | overlies 1213±23 (OxA-29285), underlies 388±27 (OxA-30803) | 8th-17th C^2^ | NB15 |
| PYS | 306 | Cf. *Rattus*/Muridae | humerus |  | Fail |  | overlies 1213±23 (OxA-29285), underlies 388±27 (OxA-30803) | 8th-17th C^2^ | NB16 |
| PYS | 306 | Cf. *Rattus*/Muridae | humerus |  | Fail |  | overlies 1213±23 (OxA-29285), underlies 388±27 (OxA-30803) | 8th-17th C^2^ | NB6 |
| PYS | 306 | Cf. *Rattus*/Muridae | humerus |  | Fail |  | overlies 1213±23 (OxA-29285), underlies 388±27 (OxA-30803) | 8th-17th C^2^ | NB7 |
| PYS | 306 | Cf. *Rattus*/Muridae | humerus |  | Fail |  | overlies 1213±23 (OxA-29285), underlies 388±27 (OxA-30803) | 8th-17th C^2^ | NB8 |
| PYS | 306 | Cf. *Rattus*/Muridae | humerus |  | Fail |  | overlies 1213±23 (OxA-29285), underlies 388±27 (OxA-30803) | 8th-17th C^2^ | NB9 |
| **Site** | **Context** | **Original Attribution^1^** | **Element** | **Dental analysis** | **ZooMS** | **aDNA** | **uncal BP date (bold = direct)** | **cal CE range** | **Specimen ID** |
| PYS | 307 | Cf. *Rattus*/Muridae | phalanx |  | *Group 1 (Mastomys sp.)* |  | overlies 1213±23 (OxA-29285), underlies 388±27 (OxA-30803) | 8th-17th C^2^ | NB100a |
| PYS | 307 | Cf. *Rattus*/Muridae | phalanx |  | *Group 1 (Mastomys sp.)* |  | overlies 1213±23 (OxA-29285), underlies 388±27 (OxA-30803) | 8th-17th C^2^ | NB100b |
| PYS | 307 | Cf. *Rattus*/Muridae | phalanx |  | *Group 1 (Mastomys sp.)* |  | overlies 1213±23 (OxA-29285), underlies 388±27 (OxA-30803) | 8th-17th C^2^ | NB100c |
| PYS | 307 | Cf. *Rattus*/Muridae | phalanx |  | *Group 1 (Mastomys sp.)* |  | overlies 1213±23 (OxA-29285), underlies 388±27 (OxA-30803) | 8th-17th C^2^ | NB100d |
| PYS | 307 | Cf. *Rattus*/Muridae | phalanx |  | *Group 1 (Mastomys sp.)* |  | overlies 1213±23 (OxA-29285), underlies 388±27 (OxA-30803) | 8th-17th C^2^ | NB100e |
| PYS | 307 | Cf. *Rattus*/Muridae | phalanx |  | *Group 2 (Gerbilliscus sp.)* |  | overlies 1213±23 (OxA-29285), underlies 388±27 (OxA-30803) | 8th-17th C^2^ | NB100f |
| PYS | 307 | Cf. *Rattus*/Muridae | phalanx |  | *Group 1 (Mastomys sp.)* |  | overlies 1213±23 (OxA-29285), underlies 388±27 (OxA-30803) | 8th-17th C^2^ | NB100g |
| PYS | 307 | Cf. *Rattus*/Muridae | phalanx |  | *Group 1 (Mastomys sp.)* |  | overlies 1213±23 (OxA-29285), underlies 388±27 (OxA-30803) | 8th-17th C^2^ | NB100h |
| PYS | 307 | Cf. *Rattus*/Muridae | phalanx |  | *Group 1 (Mastomys sp.)* |  | overlies 1213±23 (OxA-29285), underlies 388±27 (OxA-30803) | 8th-17th C^2^ | NB100i |
| PYS | 307 | Cf. *Rattus*/Muridae | phalanx |  | Fail |  | overlies 1213±23 (OxA-29285), underlies 388±27 (OxA-30803) | 8th-17th C^2^ | NB100j |
| PYS | 307 | Cf. *Rattus*/Muridae | phalanx |  | *Group 1 (Mastomys sp.)* |  | overlies 1213±23 (OxA-29285), underlies 388±27 (OxA-30803) | 8th-17th C^2^ | NB100k |
| **Site** | **Context** | **Original Attribution^1^** | **Element** | **Dental analysis** | **ZooMS** | **aDNA** | **uncal BP date (bold = direct)** | **cal CE range** | **Specimen ID** |
| PYS | 307 | Cf. *Rattus*/Muridae | phalanx |  | *Group 1 (Mastomys sp.)* |  | overlies 1213±23 (OxA-29285), underlies 388±27 (OxA-30803) | 8th-17th C^2^ | NB100l |
| PYS | 307 | Cf. *Rattus*/Muridae | radius |  | *Group 2 (Gerbilliscus sp.)* |  | overlies 1213±23 (OxA-29285), underlies 388±27 (OxA-30803) | 8th-17th C^2^ | NB101 |
| PYS | 307 | Cf. *Rattus*/Muridae | calcaneum |  | *Group 1 (Mastomys sp.)* |  | overlies 1213±23 (OxA-29285), underlies 388±27 (OxA-30803) | 8th-17th C^2^ | NB102 |
| PYS | 307 | Cf. *Rattus*/Muridae | calcaneum |  | Fail |  | overlies 1213±23 (OxA-29285), underlies 388±27 (OxA-30803) | 8th-17th C^2^ | NB103 |
| PYS | 307 | Cf. *Rattus*/Muridae | calcaneum |  | *Group 2 (Gerbilliscus sp.)* |  | overlies 1213±23 (OxA-29285), underlies 388±27 (OxA-30803) | 8th-17th C^2^ | NB104 |
| PYS | 307 | Cf. *Rattus*/Muridae | calcaneum |  | Fail |  | overlies 1213±23 (OxA-29285), underlies 388±27 (OxA-30803) | 8th-17th C^2^ | NB105 |
| PYS | 307 | Cf. *Rattus*/Muridae | femur |  | *Group 1 (Mastomys sp.)* |  | overlies 1213±23 (OxA-29285), underlies 388±27 (OxA-30803) | 8th-17th C^2^ | NB106 |
| PYS | 307 | Cf. *Rattus*/Muridae | femur |  | *Group 2 (Gerbilliscus sp.)* |  | overlies 1213±23 (OxA-29285), underlies 388±27 (OxA-30803) | 8th-17th C^2^ | NB107 |
| PYS | 307 | Cf. *Rattus*/Muridae | femur |  | Fail |  | overlies 1213±23 (OxA-29285), underlies 388±27 (OxA-30803) | 8th-17th C^2^ | NB108 |
| PYS | 307 | Cf. *Rattus*/Muridae | femur |  | Fail |  | overlies 1213±23 (OxA-29285), underlies 388±27 (OxA-30803) | 8th-17th C^2^ | NB109 |
| PYS | 307 | Cf. *Rattus*/Muridae | femur |  | Fail |  | overlies 1213±23 (OxA-29285), underlies 388±27 (OxA-30803) | 8th-17th C^2^ | NB110 |

| **Site** | **Context** | **Original Attribution^1^** | **Element** | **Dental analysis** | **ZooMS** | **aDNA** | **uncal BP date (bold = direct)** | **cal CE range** | **Specimen ID** |
| --- | --- | --- | --- | --- | --- | --- | --- | --- | --- |
| PYS | 307 | Cf. *Rattus*/Muridae | femur |  | Fail |  | overlies 1213±23 (OxA-29285), underlies 388±27 (OxA-30803) | 8th-17th C^2^ | NB111 |
| PYS | 307 | Cf. *Rattus*/Muridae | scapula |  | *Group 1 (Mastomys sp.)* |  | overlies 1213±23 (OxA-29285), underlies 388±27 (OxA-30803) | 8th-17th C^2^ | NB112 |
| PYS | 307 | Cf. *Rattus*/Muridae | maxilla |  | *Group 1 (Mastomys sp.)* |  | overlies 1213±23 (OxA-29285), underlies 388±27 (OxA-30803) | 8th-17th C^2^ | NB129 |
| PYS | 307 | Cf. *Rattus*/Muridae | maxilla |  | *Group 1 (Mastomys sp.)* |  | overlies 1213±23 (OxA-29285), underlies 388±27 (OxA-30803) | 8th-17th C^2^ | NB130 |
| PYS | 307 | Cf. *Rattus*/Muridae | mandile |  | *Group 1 (Mastomys sp.)* |  | overlies 1213±23 (OxA-29285), underlies 388±27 (OxA-30803) | 8th-17th C^2^ | NB131 |
| PYS | 307 | Cf. *Rattus*/Muridae | mandible |  | *Group 1 (Mastomys sp.)* |  | overlies 1213±23 (OxA-29285), underlies 388±27 (OxA-30803) | 8th-17th C^2^ | NB132 |
| PYS | 307 | Cf. *Rattus*/Muridae | maxilla |  | *Group 2 (Gerbilliscus sp.)* |  | overlies 1213±23 (OxA-29285), underlies 388±27 (OxA-30803) | 8th-17th C^2^ | NB133 |
| PYS | 307 | Cf. *Rattus*/Muridae | maxilla |  | *Group 1 (Mastomys sp.)* |  | overlies 1213±23 (OxA-29285), underlies 388±27 (OxA-30803) | 8th-17th C^2^ | NB134 |
| PYS | 307 | Cf. *Rattus*/Muridae | maxilla |  | *Group 1 (Mastomys sp.)* |  | overlies 1213±23 (OxA-29285), underlies 388±27 (OxA-30803) | 8th-17th C^2^ | NB135 |
| PYS | 307 | Cf. *Rattus*/Muridae | maxilla |  | *Group 2 (Gerbilliscus sp.)* |  | overlies 1213±23 (OxA-29285), underlies 388±27 (OxA-30803) | 8th-17th C^2^ | NB136 |

| **Site** | **Context** | **Original Attribution^1^** | **Element** | **Dental analysis** | **ZooMS** | **aDNA** | **uncal BP date (bold = direct)** | **cal CE range** | **Specimen ID** |
| --- | --- | --- | --- | --- | --- | --- | --- | --- | --- |
| PYS | 307 | Cf. *Rattus*/Muridae | maxilla |  | *Group 1 (Mastomys sp.)* |  | overlies 1213±23 (OxA-29285), underlies 388±27 (OxA-30803) | 8th-17th C^2^ | NB137 |
| PYS | 307 | Cf. *Rattus*/Muridae | maxilla |  | Fail |  | overlies 1213±23 (OxA-29285), underlies 388±27 (OxA-30803) | 8th-17th C^2^ | NB138 |
| PYS | 307 | Cf. *Rattus*/Muridae | maxilla |  | *Group 2 (Gerbilliscus sp.)* |  | overlies 1213±23 (OxA-29285), underlies 388±27 (OxA-30803) | 8th-17th C^2^ | NB139 |
| PYS | 307 | Cf. *Rattus*/Muridae | maxilla |  | *Group 1 (Mastomys sp.)* |  | overlies 1213±23 (OxA-29285), underlies 388±27 (OxA-30803) | 8th-17th C^2^ | NB140 |
| PYS | 307 | Cf. *Rattus*/Muridae | maxilla |  | *Group 1 (Mastomys sp.)* |  | overlies 1213±23 (OxA-29285), underlies 388±27 (OxA-30803) | 8th-17th C^2^ | NB141 |
| PYS | 307 | Cf. *Rattus*/Muridae | maxilla |  | *Group 1 (Mastomys sp.)* |  | overlies 1213±23 (OxA-29285), underlies 388±27 (OxA-30803) | 8th-17th C^2^ | NB142 |
| PYS | 307 | Cf. *Rattus*/Muridae | mandible |  | Fail |  | overlies 1213±23 (OxA-29285), underlies 388±27 (OxA-30803) | 8th-17th C^2^ | NB155 |
| PYS | 307 | Cf. *Rattus*/Muridae | mandible | Not *Rattus* | *Group 1 (Mastomys sp.)* |  | overlies 1213±23 (OxA-29285), underlies 388±27 (OxA-30803) | 8th-17th C^2^ | NB157 |
| PYS | 307 | Cf. *Rattus*/Muridae | mandible | Not *Rattus* | *Group 1 (Mastomys sp.)* |  | overlies 1213±23 (OxA-29285), underlies 388±27 (OxA-30803) | 8th-17th C^2^ | NB158 |
| PYS | 307 | Cf. *Rattus*/Muridae | mandible | Not *Rattus* | *Group 1 (Mastomys sp.)* |  | overlies 1213±23 (OxA-29285), underlies 388±27 (OxA-30803) | 8th-17th C^2^ | NB159 |

| **Site** | **Context** | **Original Attribution^1^** | **Element** | **Dental analysis** | **ZooMS** | **aDNA** | **uncal BP date (bold = direct)** | **cal CE range** | **Specimen ID** |
| --- | --- | --- | --- | --- | --- | --- | --- | --- | --- |
| PYS | 307 | Cf. *Rattus*/Muridae | mandible | Not *Rattus* | *Group 1 (Mastomys sp.)* |  | overlies 1213±23 (OxA-29285), underlies 388±27 (OxA-30803) | 8th-17th C^2^ | NB160 |
| PYS | 307 | Cf. *Rattus*/Muridae | mandible |  | *Group 1 (Mastomys sp.)* |  | overlies 1213±23 (OxA-29285), underlies 388±27 (OxA-30803) | 8th-17th C^2^ | NB163 |
| PYS | 307 | Cf. *Rattus*/Muridae | mandible |  | Fail |  | overlies 1213±23 (OxA-29285), underlies 388±27 (OxA-30803) | 8th-17th C^2^ | NB165 |
| PYS | 307 | Cf. *Rattus*/Muridae | incisor |  | *Group 1 (Mastomys sp.)* |  | overlies 1213±23 (OxA-29285), underlies 388±27 (OxA-30803) | 8th-17th C^2^ | NB175a |
| PYS | 307 | Cf. *Rattus*/Muridae | incisor |  | *Group 1 (Mastomys sp.)* |  | overlies 1213±23 (OxA-29285), underlies 388±27 (OxA-30803) | 8th-17th C^2^ | NB175b |
| PYS | 307 | Cf. *Rattus*/Muridae | humerus |  | *R. rattus* |  | overlies 1213±23 (OxA-29285), underlies 388±27 (OxA-30803) | 8th-17th C^2^ | NB65 |
| PYS | 307 | Cf. *Rattus*/Muridae | humerus |  | *Group 2 (Gerbilliscus sp.)* |  | overlies 1213±23 (OxA-29285), underlies 388±27 (OxA-30803) | 8th-17th C^2^ | NB66 |
| PYS | 307 | Cf. *Rattus*/Muridae | mandible |  | *Group 1 (Mastomys sp.)* |  | overlies 1213±23 (OxA-29285), underlies 388±27 (OxA-30803) | 8th-17th C^2^ | NB67 |
| PYS | 307 | Cf. *Rattus*/Muridae | tibia |  | *Group 2 (Gerbilliscus sp.)* |  | overlies 1213±23 (OxA-29285), underlies 388±27 (OxA-30803) | 8th-17th C^2^ | NB70 |
| PYS | 307 | Cf. *Rattus*/Muridae | tibia |  | *Group 2 (Gerbilliscus sp.)* |  | overlies 1213±23 (OxA-29285), underlies 388±27 (OxA-30803) | 8th-17th C^2^ | NB71 |
| PYS | 307 | Cf. *Rattus*/Muridae | tibia |  | *Group 1 (Mastomys sp.)* |  | overlies 1213±23 (OxA-29285), underlies 388±27 (OxA-30803) | 8th-17th C^2^ | NB72 |
| **Site** | **Context** | **Original Attribution^1^** | **Element** | **Dental analysis** | **ZooMS** | **aDNA** | **uncal BP date (bold = direct)** | **cal CE range** | **Specimen ID** |
| PYS | 307 | Cf. *Rattus*/Muridae | tibia |  | *Group 2 (Gerbilliscus sp.)* |  | overlies 1213±23 (OxA-29285), underlies 388±27 (OxA-30803) | 8th-17th C^2^ | NB73 |
| PYS | 307 | Cf. *Rattus*/Muridae | tibia |  | *Group 1 (Mastomys sp.)* |  | overlies 1213±23 (OxA-29285), underlies 388±27 (OxA-30803) | 8th-17th C^2^ | NB74 |
| PYS | 307 | Cf. *Rattus*/Muridae | tibia |  | *Group 1 (Mastomys sp.)* |  | overlies 1213±23 (OxA-29285), underlies 388±27 (OxA-30803) | 8th-17th C^2^ | NB75 |
| PYS | 307 | Cf. *Rattus*/Muridae | tibia |  | Fail |  | overlies 1213±23 (OxA-29285), underlies 388±27 (OxA-30803) | 8th-17th C^2^ | NB76 |
| PYS | 307 | Cf. *Rattus*/Muridae | tibia |  | *Group 1 (Mastomys sp.)* |  | overlies 1213±23 (OxA-29285), underlies 388±27 (OxA-30803) | 8th-17th C^2^ | NB77 |
| PYS | 307 | Cf. *Rattus*/Muridae | tibia |  | *Group 1 (Mastomys sp.)* |  | overlies 1213±23 (OxA-29285), underlies 388±27 (OxA-30803) | 8th-17th C^2^ | NB78 |
| PYS | 307 | Cf. *Rattus*/Muridae | tibia |  | *Group 1 (Mastomys sp.)* |  | overlies 1213±23 (OxA-29285), underlies 388±27 (OxA-30803) | 8th-17th C^2^ | NB79 |
| PYS | 307 | Cf. *Rattus*/Muridae | tibia |  | *Group 1 (Mastomys sp.)* |  | overlies 1213±23 (OxA-29285), underlies 388±27 (OxA-30803) | 8th-17th C^2^ | NB80 |
| PYS | 307 | Cf. *Rattus*/Muridae | tibia |  | *Group 1 (Mastomys sp.)* |  | overlies 1213±23 (OxA-29285), underlies 388±27 (OxA-30803) | 8th-17th C^2^ | NB81 |
| PYS | 307 | Cf. *Rattus*/Muridae | tibia |  | *Group 1 (Mastomys sp.)* |  | overlies 1213±23 (OxA-29285), underlies 388±27 (OxA-30803) | 8th-17th C^2^ | NB82 |
| PYS | 307 | Cf. *Rattus*/Muridae | tibia |  | Fail |  | overlies 1213±23 (OxA-29285), underlies 388±27 (OxA-30803) | 8th-17th C^2^ | NB83 |
| **Site** | **Context** | **Original Attribution^1^** | **Element** | **Dental analysis** | **ZooMS** | **aDNA** | **uncal BP date (bold = direct)** | **cal CE range** | **Specimen ID** |
| PYS | 307 | Cf. *Rattus*/Muridae | femur |  | Fail |  | overlies 1213±23 (OxA-29285), underlies 388±27 (OxA-30803) | 8th-17th C^2^ | NB84 |
| PYS | 307 | Cf. *Rattus*/Muridae | phalanx |  | Fail |  | overlies 1213±23 (OxA-29285), underlies 388±27 (OxA-30803) | 8th-17th C^2^ | NB99a |
| PYS | 307 | Cf. *Rattus*/Muridae | phalanx |  | *Group 1 (Mastomys sp.)* |  | overlies 1213±23 (OxA-29285), underlies 388±27 (OxA-30803) | 8th-17th C^2^ | NB99b |
| PYS | 307 | Cf. *Rattus*/Muridae | phalanx |  | Fail |  | overlies 1213±23 (OxA-29285), underlies 388±27 (OxA-30803) | 8th-17th C^2^ | NB99c |
| PYS | 307 | Cf. *Rattus*/Muridae | phalanx |  | *Group 1 (Mastomys sp.)* |  | overlies 1213±23 (OxA-29285), underlies 388±27 (OxA-30803) | 8th-17th C^2^ | NB99d |
| PYS | 307 | Cf. *Rattus*/Muridae | phalanx |  | *Group 1 (Mastomys sp.)* |  | overlies 1213±23 (OxA-29285), underlies 388±27 (OxA-30803) | 8th-17th C^2^ | NB99e |
| PYS | 307 | Cf. *Rattus*/Muridae | phalanx |  | Fail |  | overlies 1213±23 (OxA-29285), underlies 388±27 (OxA-30803) | 8th-17th C^2^ | NB99f |
| PYS | 307 | Cf. *Rattus*/Muridae | phalanx |  | *Group 1 (Mastomys sp.)* |  | overlies 1213±23 (OxA-29285), underlies 388±27 (OxA-30803) | 8th-17th C^2^ | NB99g |
| PYS | 307 | Cf. *Rattus*/Muridae | phalanx |  | *Group 1 (Mastomys sp.)* |  | overlies 1213±23 (OxA-29285), underlies 388±27 (OxA-30803) | 8th-17th C^2^ | NB99h |
| PYS | 307 | Cf. *Rattus*/Muridae | phalanx |  | Fail |  | overlies 1213±23 (OxA-29285), underlies 388±27 (OxA-30803) | 8th-17th C^2^ | NB99i |
| PYS | 307 | Cf. *Rattus*/Muridae | phalanx |  | Fail |  | overlies 1213±23 (OxA-29285), underlies 388±27 (OxA-30803) | 8th-17th C^2^ | NB99j |
| **Site** | **Context** | **Original Attribution^1^** | **Element** | **Dental analysis** | **ZooMS** | **aDNA** | **uncal BP date (bold = direct)** | **cal CE range** | **Specimen ID** |
| PYS | 307 | Cf. *Rattus*/Muridae | phalanx |  | *Group 1 (Mastomys sp.)* |  | overlies 1213±23 (OxA-29285), underlies 388±27 (OxA-30803) | 8th-17th C^2^ | NB99k |
| PYS | 307 | Cf. *Rattus*/Muridae | phalanx |  | Fail |  | overlies 1213±23 (OxA-29285), underlies 388±27 (OxA-30803) | 8th-17th C^2^ | NB99l |
| MTSE | TPO1(3) | *R. rattus* | femur |  | *R. rattus* |  | **448±20 (Wk-41391)** | **1435–1490** | NB54 |
| SC | 203 | Rat | various |  |  | *R. rattus* | **348±20 (Wk-43701)** | **1495–1640** | RAT_05 |
| SC | 203 | Rat | various |  |  | Fail | **233±20 (Wk-43702)** | **1650–1800** | RAT_06 |
| PMZ | 1/008 | Muridae | tibia |  | Fail |  | none | 7th C or later | NB1 |
| PMZ | 203 | Muridae Aff. *Rattus* | mandible | Not *Rattus* | *Group 2 (Gerbilliscus sp.)* |  | none | 7th-14th C^2^ | NB63 |
| MBYN | TP01(11) | *R. rattus* | limb bone |  |  | Not *Rattus* | overlies 350±50 (Pta-7965) | 15th-17th C or later | RAT_08 |
| MBYN | TP01(11) | *R. rattus* | limb bone |  |  | Fail | overlies 350±50 (Pta-7965) | 15th-17th C or later | RAT_16 |
| CHO | TP04(13) | *R. rattus* | pelvis |  |  | Not *Rattus* | overlies 1180±60 (Pta-7978) | 8th-11th C or later | RAT_15 |
| CHO | TP04(14) | *R. rattus* | femur |  |  | Fail | overlies 1180±60 (Pta-7978) | 8th-11th C or later | RAT_09 |
| CHO | TP04(18) | *R. rattus* | long bone |  | *Group 2 (Gerbilliscus sp.)* |  | assoc. with 1180±60 (Pta-7978) | 8th-11th C | RHE.CHO |
| VMB | 12002 | Rat | mandible |  | *Group 2 (Gerbilliscus sp.)* |  | none; single occupation layer | 15th C^3^ | NB86 |
| VMB | 12002 | Rat | vertebra |  | *Group 2 (Gerbilliscus sp.)* |  | none; single occupation layer | 15th C^3^ | NB52 |
| VMB | 12002 | Rat | long bone |  |  | *R. rattus* | none; single occupation layer | 15th C^3^ | RAT_07 |
| VMB | 12002 | Rat | scapula |  | *Group 2 (Gerbilliscus sp.)* |  | none; single occupation layer | 15th C^3^ | NB50 |
| VMB | 12002 | Rat | pelvis |  | *Group 2 (Gerbilliscus sp.)* |  | none; single occupation layer | 15th C^3^ | NB51 |
| PK | 203 | *R. rattus* | right dentary | *R. rattus* | *R. rattus* |  | **1075 ±20 (Wk-41874)** | **980–1025** | NB171 |
| PK | 203 | *R. rattus* | tibia |  | *R. rattus* |  | assoc. with 1075±20 (Wk-41874), overlies 873±21 (Wk-40973) | 7th?/10th-12th C or later | NB114 |
| PK | 203 | *R. rattus* | femur |  | *R. rattus* |  | assoc. with 1075±20 (Wk-41874), overlies 873±21 (Wk-40973) | 7th?/10th-12th C or later | NB122 |

| **Site** | **Context** | **Original Attribution^1^** | **Element** | **Dental analysis** | **ZooMS** | **aDNA** | **uncal BP date (bold = direct)** | **cal CE range** | **Specimen ID** |
| --- | --- | --- | --- | --- | --- | --- | --- | --- | --- |
| PK | 203 | *R. rattus* | tibia |  | *R. rattus* |  | assoc. with 1075±20 (Wk-41874), overlies 873±21 (Wk-40973) | 7th?/10th-12th C or later | NB123 |
| PK | 203 | *R. rattus* | femur |  | *R. rattus* |  | assoc. with 1075±20 (Wk-41874), overlies 873±21 (Wk-40973) | 7th?/10th-12th C or later | NB124 |
| PK | 203 | *R. rattus* | humerus |  | *R. rattus* |  | assoc. with 1075±20 (Wk-41874), overlies 873±21 (Wk-40973) | 7th?/10th-12th C or later | NB125 |
| PK | 203 | *R. rattus* | femur |  | *R. rattus* |  | assoc. with 1075±20 (Wk-41874), overlies 873±21 (Wk-40973) | 7th?/10th-12th C or later | NB127 |
| PK | 203 | *R. rattus* | maxilla |  | *R. rattus* |  | assoc. with 1075±20 (Wk-41874), overlies 873±21 (Wk-40973) | 7th?/10th-12th C or later | NB150 |
| PK | 203 | *R. rattus* | maxilla |  | *R. rattus* |  | assoc. with 1075±20 (Wk-41874), overlies 873±21 (Wk-40973) | 7th?/10th-12th C or later | NB152 |
| PK | 203 | *R. rattus* | maxilla |  | *R. rattus* |  | assoc. with 1075±20 (Wk-41874), overlies 873±21 (Wk-40973) | 7th?/10th-12th C or later | NB153 |
| PK | 203 | *R. rattus* | left dentary |  | *R. rattus* |  | assoc. with 1075±20 (Wk-41874), overlies 873±21 (Wk-40973) | 7th?/10th-12th C or later | NB170 |
| PK | 203 | *R. rattus* | right dentary | *R. rattus* | *R. rattus* |  | assoc. with 1075±20 (Wk-41874), overlies 873±21 (Wk-40973) | 7th?/10th-12th C or later | NB173 |
| PK | 404 | *R. rattus* | humerus |  | *R. rattus* |  | assoc. with 994±20 (Wk-41877), 998±20 (Wk-41875), 533±20 (Wk-41842) | 7th?/10th-12th C or later | NB115 |
| PK | 404 | *R. rattus* | left dentary |  | *R. rattus* |  | **994±20 (Wk-41877)** | **1020–1150** | NB154 |
| PK | 404 | *R. rattus* | femur |  | *R. rattus* |  | **998±20 (Wk-41875)** | **1020–1150** | NB119 |
| PK | 404 | *R. rattus* | femur |  | *R. rattus* |  | assoc. with 994±20 (Wk-41877), 998±20 (Wk-41875), 533±20 (Wk-41842) | 7th?/10th-12th C or later | NB120 |
| PK | 404 | *R. rattus* | tibia |  | *R. rattus* |  | assoc. with 994±20 (Wk-41877), 998±20 (Wk-41875), 533±20 (Wk-41842) | 7th?/10th-12th C or later | NB121 |

| **Site** | **Context** | **Original Attribution^1^** | **Element** | **Dental analysis** | **ZooMS** | **aDNA** | **uncal BP date (bold = direct)** | **cal CE range** | **Specimen ID** |
| --- | --- | --- | --- | --- | --- | --- | --- | --- | --- |
| PK | 404 | *R. rattus* | maxilla |  | *R. rattus* |  | assoc. with 994±20 (Wk-41877), 998±20 (Wk-41875), 533±20 (Wk-41842) | 7th?/10th-12th C or later | NB149 |
| PK | 404 | *R. rattus* | right dentary | *R. rattus* | *R. rattus* |  | assoc. with 994±20 (Wk-41877), 998±20 (Wk-41875), 533±20 (Wk-41842) | 7th?/10th-12th C or later | NB168 |
| PK | 404 | *R. rattus* | left dentary | *R. rattus* | *R. rattus* |  | assoc. with 994±20 (Wk-41877), 998±20 (Wk-41875), 533±20 (Wk-41842) | 7th?/10th-12th C or later | NB169 |
| PK | 405 | *R. rattus* | tibia |  | *R. rattus* |  | **1271±20 (Wk-41876)** | **685–875** | NB116 |
| PK | 405 | *R. rattus* | pelvis |  | *R. rattus* |  | assoc. with 1271±20 (Wk-41876) | 7th-9th C | NB117 |
| PK | 405 | *R. rattus* | femur |  | *R. rattus* |  | assoc. with 1271±20 (Wk-41876) | 7th-9th C | NB118 |
| PK | 405 | *R. rattus* | left dentary |  | *R. rattus* |  | assoc. with 1271±20 (Wk-41876) | 7th-9th C | NB167 |
| PK | 406 | *R. rattus* | vertebra |  | Group 4 (Unknown; Not *Rattus)* |  | assoc. with 1281±20 (Wk-41878) and 1220±23 (Wk-40971) | 7th-9th C | NB113 |
| PK | 406 | *R. rattus* | right dentary | *R. rattus* | *R. rattus* |  | assoc. with 1281±20 (Wk-41878) and 1220±23 (Wk-40971) | 7th-9th C | NB161 |
| PK | 406 | *R. rattus* | right dentary | *R. rattus* | *R. rattus* |  | **1281±20 (Wk-41878)** | **680–865** | NB162 |
| PK | 408 | *R. rattus* | maxilla |  | *R. rattus* |  | none | indeterminate | NB151 |
| PK | 504 | *R. rattus* | femur |  | *R. rattus* |  | none | c. 10th C or later | NB126 |
| PK | 504 | *R. rattus* | right dentary | *R. rattus* | *R. rattus* |  | none | c. 10th C or later | NB172 |
| FK | 11/006 | Sm Rodent | incisor |  | *R. rattus* |  | none | 7th-15th C^2^ | NB200 |
| FK | 12/003 | *R. rattus* | humerus |  |  | *R. rattus* | overlies 1325±23 (OxA-31426) | 7th-8th C or later | RAT_61 |
| UU | 1/07 | R. rattus | femur |  | *R. rattus* | Fail | **1611 ± 20 BP (Wk-41261)** | **421-535** | RAT_14, NB57 |
| UU | 11/004 | Sm Rodent | humerus |  | *R. rattus* |  | direct date failed; assoc. with 1178±25 (OxA-27517) | 8th-10th C or later | NB48 |
| UU | 11/006 | Sm Rodent | femur |  | *R. rattus* |  | assoc. with 1266±35 (OxA-X-2554-12) | 7th-9th C | NB201 |
| **Site** | **Context** | **Original Attribution^1^** | **Element** | **Dental analysis** | **ZooMS** | **aDNA** | **uncal BP date (bold = direct)** | **cal CE range** | **Specimen ID** |
| UU | 11/006 | Sm Rodent | tibia |  | *R. rattus* |  | assoc. with 1266±35 (OxA-X-2554-12) | 7th-9th C | NB202 |
| UU | 11/014 | Sm Rodent | femur |  | *R. rattus* |  | assoc. with 1280±26 (OxA-27515) | 7th-9th C | NB213 |
| UU | 1403 | Sm Rodent | tibia |  | *Group 1 (Mastomys sp.)* |  | overlies 1151±26 (OxA-27520) | 9th-10th C or later | NB219 |
| UU | 1404 | Sm Rodent | femur |  | *Group 1 (Mastomys sp.)* |  | assoc. with 1151±26 (OxA-27520) | 9th-10th C or later | NB208 |
| UU | 1404 | Sm Rodent | femur |  | *Group 1 (Mastomys sp.)* |  | assoc. with 1151±26 (OxA-27520) | 9th-10th C or later | NB207 |
| UU | 1404 | Sm Rodent | femur |  | *Group 1 (Mastomys sp.)* |  | assoc. with 1151±26 (OxA-27520) | 9th-10th C or later | NB206 |
| UU | 1404 | Sm Rodent | femur |  | Group 5 (Unknown; Not *Rattus)* |  | assoc. with 1151±26 (OxA-27520) | 9th-10th C or later | NB205 |
| UU | 1404 | Sm Rodent | tibia |  | *Group 1 (Mastomys sp.)* |  | assoc. with 1151±26 (OxA-27520) | 9th-10th C or later | NB210 |
| UU | 1404 | Sm Rodent | femur |  | *Group 1 (Mastomys sp.)* |  | overlies 1226±25 (OxA-27698), underlies 1151±26 (OxA-27520) | 8th-10th C | NB211 |
| UU | 1408 | Sm Rodent | femur |  | Group 5 (Unknown; Not *Rattus)* |  | overlies 1226±25 (OxA-27698), underlies 1151±26 (OxA-27520) | 8th-10th C | NB220 |
| UU | 1408 | Sm Rodent | femur |  | *Group 1 (Mastomys sp.)* |  | overlies 1226±25 (OxA-27698), underlies 1151±26 (OxA-27520) | 8th-10th C | NB245 |
| UU | 1408 | Sm Rodent | femur |  | *Group 1 (Mastomys sp.)* |  | overlies 1226±25 (OxA-27698), underlies 1151±26 (OxA-27520) | 8th-10th C | NB221 |
| UU | 1408 | Sm Rodent | femur |  | *Group 1 (Mastomys sp.)* |  | overlies 1226±25 (OxA-27698), underlies 1151±26 (OxA-27520) | 8th-10th C | NB222 |
| UU | 1408 | Sm Rodent | femur |  | *Group 1 (Mastomys sp.)* |  | overlies 1226±25 (OxA-27698), underlies 1151±26 (OxA-27520) | 8th-10th C | NB223 |
| UU | 1408 | Sm Rodent | femur |  | *Group 1 (Mastomys sp.)* |  | overlies 1226±25 (OxA-27698), underlies 1151±26 (OxA-27520) | 8th-10th C | NB224 |
| **Site** | **Context** | **Original Attribution^1^** | **Element** | **Dental analysis** | **ZooMS** | **aDNA** | **uncal BP date (bold = direct)** | **cal CE range** | **Specimen ID** |
| UU | 1408 | Sm Rodent | femur |  | *Group 1 (Mastomys sp.)* |  | overlies 1226±25 (OxA-27698), underlies 1151±26 (OxA-27520) | 8th-10th C | NB225 |
| UU | 1408 | Sm Rodent | femur |  | *Group 1 (Mastomys sp.)* |  | overlies 1226±25 (OxA-27698), underlies 1151±26 (OxA-27520) | 8th-10th C | NB226 |
| UU | 1408 | Sm Rodent | femur |  | *Group 1 (Mastomys sp.)* |  | overlies 1226±25 (OxA-27698), underlies 1151±26 (OxA-27520) | 8th-10th C | NB227 |
| UU | 1408 | Sm Rodent | femur |  | *Group 1 (Mastomys sp.)* |  | overlies 1226±25 (OxA-27698), underlies 1151±26 (OxA-27520) | 8th-10th C | NB228 |
| UU | 1408 | Sm Rodent | femur |  | *Group 1 (Mastomys sp.)* |  | overlies 1226±25 (OxA-27698), underlies 1151±26 (OxA-27520) | 8th-10th C | NB229 |
| UU | 1408 | Sm Rodent | pelvis |  | *Group 1 (Mastomys sp.)* |  | overlies 1226±25 (OxA-27698), underlies 1151±26 (OxA-27520) | 8th-10th C | NB230 |
| UU | 1408 | Sm Rodent | pelvis |  | *Group 1 (Mastomys sp.)* |  | overlies 1226±25 (OxA-27698), underlies 1151±26 (OxA-27520) | 8th-10th C | NB231 |
| UU | 1408 | Sm Rodent | pelvis |  | Group 6 (Unknown; Not *Rattus*; unlikely Rodentia) |  | overlies 1226±25 (OxA-27698), underlies 1151±26 (OxA-27520) | 8th-10th C | NB232 |
| UU | 1408 | Sm Rodent | pelvis |  | *Group 1 (Mastomys sp.)* |  | overlies 1226±25 (OxA-27698), underlies 1151±26 (OxA-27520) | 8th-10th C | NB233 |
| UU | 1408 | Sm Rodent | humerus |  | *Group 1 (Mastomys sp.)* |  | overlies 1226±25 (OxA-27698), underlies 1151±26 (OxA-27520) | 8th-10th C | NB234 |
| UU | 1408 | Sm Rodent | humerus |  | *Group 1 (Mastomys sp.)* |  | overlies 1226±25 (OxA-27698), underlies 1151±26 (OxA-27520) | 8th-10th C | NB235 |
| **Site** | **Context** | **Original Attribution^1^** | **Element** | **Dental analysis** | **ZooMS** | **aDNA** | **uncal BP date (bold = direct)** | **cal CE range** | **Specimen ID** |
| UU | 1408 | Sm Rodent | humerus |  | *Group 1 (Mastomys sp.)* |  | overlies 1226±25 (OxA-27698), underlies 1151±26 (OxA-27520) | 8th-10th C | NB236 |
| UU | 1408 | Sm Rodent | humerus |  | *Group 1 (Mastomys sp.)* |  | overlies 1226±25 (OxA-27698), underlies 1151±26 (OxA-27520) | 8th-10th C | NB238 |
| UU | 1408 | Sm Rodent | radius |  | Group 5 (Unknown; Not *Rattus)* |  | overlies 1226±25 (OxA-27698), underlies 1151±26 (OxA-27520) | 8th-10th C | NB239 |
| UU | 1408 | Sm Rodent | tibia |  | *Group 1 (Mastomys sp.)* |  | overlies 1226±25 (OxA-27698), underlies 1151±26 (OxA-27520) | 8th-10th C | NB240 |
| UU | 1408 | Sm Rodent | tibia |  | *Group 1 (Mastomys sp.)* |  | overlies 1226±25 (OxA-27698), underlies 1151±26 (OxA-27520) | 8th-10th C | NB241 |
| UU | 1408 | Sm Rodent | tibia |  | *Group 1 (Mastomys sp.)* |  | overlies 1226±25 (OxA-27698), underlies 1151±26 (OxA-27520) | 8th-10th C | NB242 |
| UU | 1408 | Sm Rodent | tibia |  | *Group 1 (Mastomys sp.)* |  | overlies 1226±25 (OxA-27698), underlies 1151±26 (OxA-27520) | 8th-10th C | NB243 |
| UU | 1408 | Sm Rodent | tibia |  | *Group 1 (Mastomys sp.)* |  | overlies 1226±25 (OxA-27698), underlies 1151±26 (OxA-27520) | 8th-10th C | NB244 |
| UU | 1412 | Sm Rodent | femur |  | *Group 1 (Mastomys sp.)* |  | overlies 1226±25 (OxA-27698), underlies 1151±26 (OxA-27520) | 8th-10th C | NB209 |
| UU | 1423 | Cf. *R. rattus* | mandible | *R. rattus* | *R. rattus* |  | **1482±20 BP (Wk-41871)** | **580–645** | NB68 |
| UU | 1423 | Muridae | femur |  | *R. rattus* |  | assoc. with 1482±20 (Wk-41871) and 1413±20 BP (Wk-41873) | 7th-8th C | NB61 |
| UU | 1423 | Cf. *R. rattus* | mandible | *R. rattus* | *R. rattus* |  | **1413±20 BP (Wk-41873)** | **635–675** | NB62 |
| **Site** | **Context** | **Original Attribution^1^** | **Element** | **Dental analysis** | **ZooMS** | **aDNA** | **uncal BP date (bold = direct)** | **cal CE range** | **Specimen ID** |
| UU | 1428 | Cf. *R. rattus* | mandible | *R. rattus* | *R. rattus* |  | direct date failed; underlies 1413±20 (Wk-41873), 1482±20 (Wk-41871) | 7th-8th C | NB64 |
| UU | 1428 | Sm Rodent | limb bone |  | *R. rattus* |  | underlies 1413±20 (Wk-41873), 1482±20 (Wk-41871) | 7th-8th C | NB214 |
| UU | 1431 | Muridae | tibia |  | *R. rattus* |  | direct date failed; overlies 1265±23 (OxA-28189) | 7th-9th C | NB60 |
| UU | 1501 | Micromammal | tibia |  | *Group 1 (Mastomys sp.)* |  | overlies 1265±45 (OxA-30955) | 7th-9th C or later | NB212 |
| UU | 1507 | Sm Rodent | femur |  | *Group 1 (Mastomys sp.)* |  | overlies 1265±45 (OxA-30955) | 7th-9th C or later | NB204 |
| UU | 1507 | Sm Rodent | tibia |  | *Group 1 (Mastomys sp.)* |  | overlies 1265±45 (OxA-30955) | 7th-9th C or later | NB203 |
| UU | 1509 | Micromammal | tibia |  | Group 5 (Unknown; Not *Rattus)* |  | overlies 1265±45 (OxA-30955) | 7th-9th C or later | NB218 |
| UU | 1511 | Sm Rodent | mandible |  | *R. rattus* |  | overlies 1265±45 (OxA-30955) | 7th-9th C or later | NB215 |
| UU | 1511 | Sm Rodent | femur |  | *R. rattus* |  | overlies 1265±45 (OxA-30955) | 7th-9th C or later | NB216 |
| UU | 1557 | Sm Rodent | femur |  | *R. rattus* |  | underlies 1265±45 (OxA-30955) | 7th-9th C | NB217 |
| SM | 10020 | Rat | long bone |  |  | *R. rattus* | none; single occupation layer | 15th C^3^ | RAT_02 |
| SM | 10035 | Rat | pelvis |  |  | *R. rattus* | none; single occupation layer | 15th C^3^ | RAT_03 |
| SM | 10024 | Rat | mandible |  |  | *R. rattus* | none; single occupation layer | 15th C^3^ | RAT_59 |
| SM | 10026 | Rat | tibia |  |  | *R. rattus* | none; single occupation layer | 15th C^3^ | RAT_46 |
| SM | 10002 | Rat | tibia |  |  | Not *Rattus* | none; single occupation layer | 15th C^3^ | RAT_60 |
| SM | 10020 | Rat | femur |  | *R. rattus* |  | none; single occupation layer | 15th C^3^ | NB55 |
| SM | 10020 | Rat | long bone |  |  | *R. rattus* | none; single occupation layer | 15th C^3^ | RAT_44 |
| SM | 10020 | Rat | tibia |  |  | *R. rattus* | none; single occupation layer | 15th C^3^ | RAT_57 |
| SM | 10035 | Rat | femur |  |  | *R. rattus* | none; single occupation layer | 15th C^3^ | RAT_10 |
| **Site** | **Context** | **Original Attribution^1^** | **Element** | **Dental analysis** | **ZooMS** | **aDNA** | **uncal BP date (bold = direct)** | **cal CE range** | **Specimen ID** |
| SM | 13007 | Rat | long bone |  |  | *R. rattus* | none; single occupation layer | 15th C^3^ | RAT_58 |
| SM | 15003 | Rat | femur |  | *R. rattus* |  | **582±20 (Wk-41390)** | **1320-1425** | NB56 |
| SM | 15009 | Rat | humerus |  |  | Fail | none; single occupation layer | 15th C^3^ | RAT_11 |
| SM | 15010 | Rat | humerus |  |  | *R. rattus* | none; single occupation layer | 15th C^3^ | RAT_45 |
| SMA | 12/13 | Muridae | calcaneum |  | *R. rattus* |  | assoc. with 1163±23 (OxA-30709), 1276±24 (OxA-30708) | 7th-10th C | NB49 |
| DMB | niv. IX | *R. rattus* | humerus |  | *R. rattus* |  | direct date failed | 8th-10th C | NB58 |
