# Supplementary figures and images for: Reconstructing Asian faunal introductions to eastern Africa from multi-proxy biomolecular and archaeological datasets
Source: PLoS One. 2017 Aug 17;12(8):e0182565. doi: 10.1371/journal.pone.0182565 (PMC5560628; doi:10.1371/journal.pone.0182565)

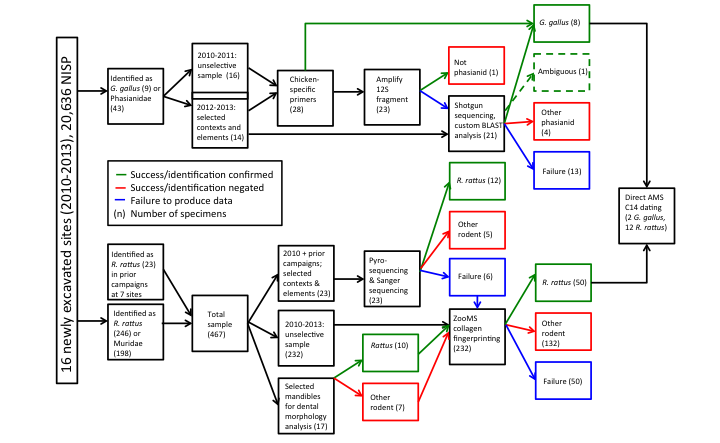

Supplement: S1 Fig — Tree illustrates the selection of faunal samples, the order in which specific analyses were applied to each subsample, and result. (TIF) [file pone.0182565.s010.tif]

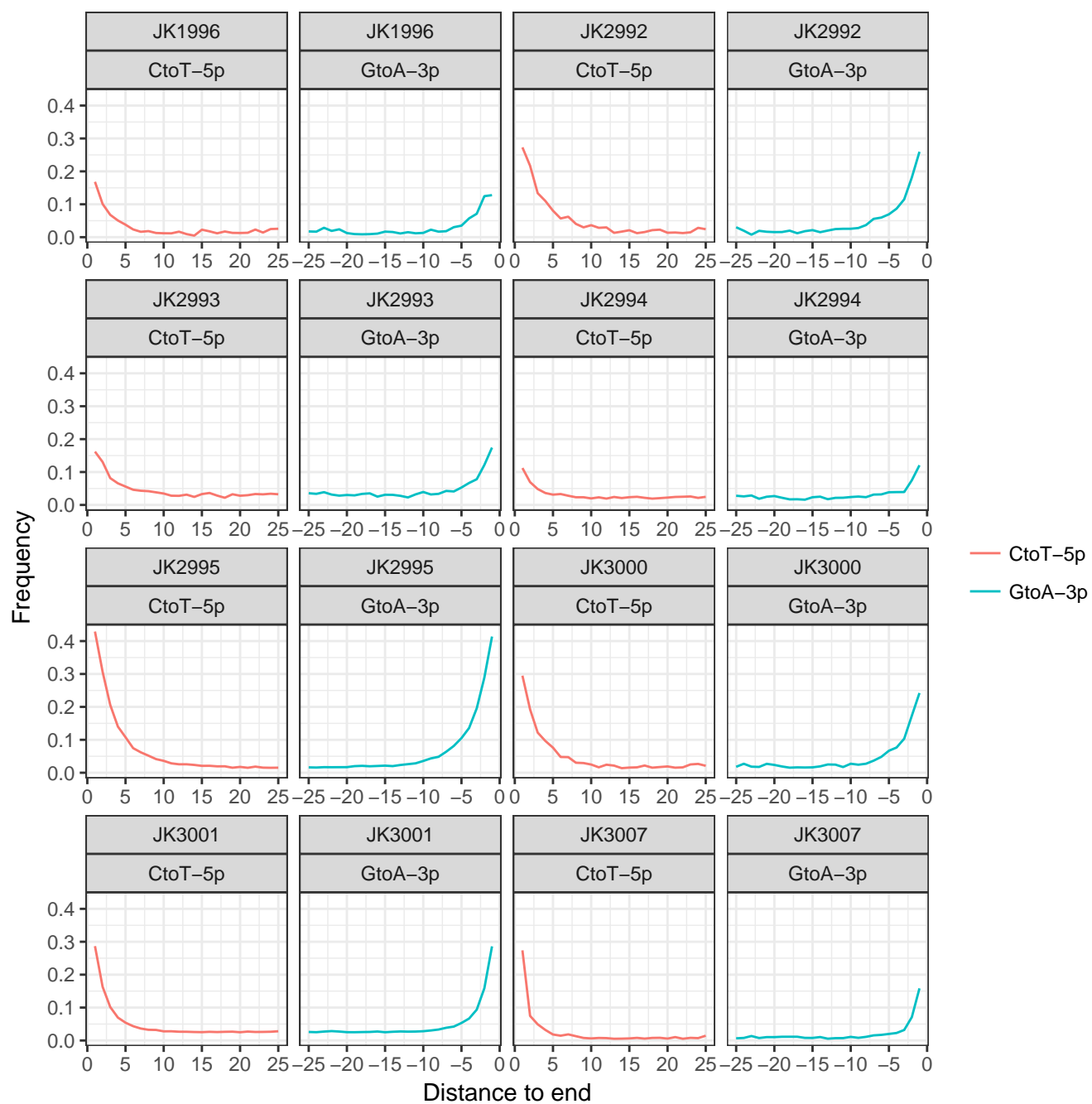

Supplement: S2 Fig — For each of the sequenced specimens (specimen numbers indicated by JK0000), mapDamage analysis illustrates C to T (red) and G to A (blue) frequencies of mis-incorporation at 3’ and 5’ ends. (PDF) [file pone.0182565.s011.pdf]

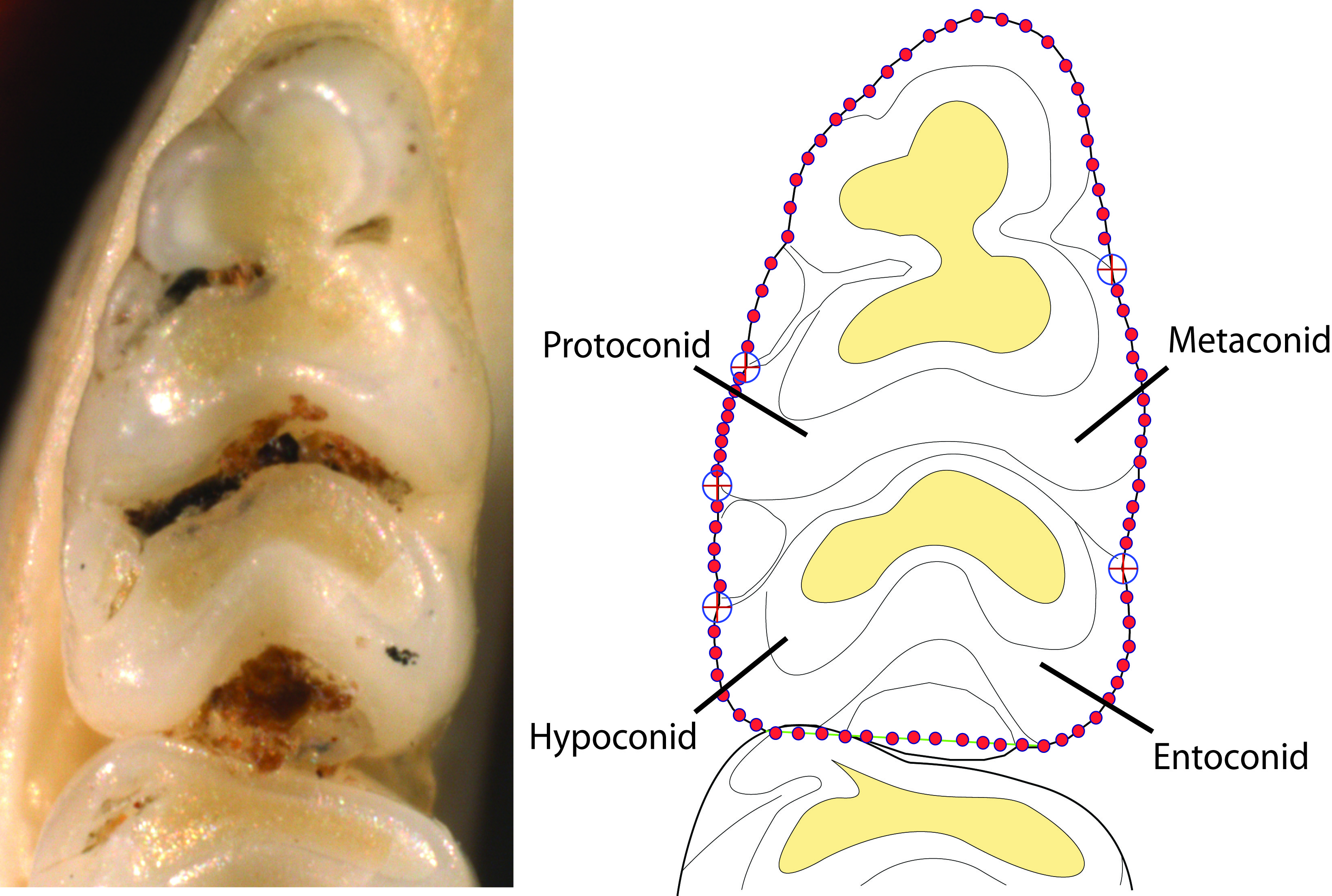

Supplement: S3 Fig — R. exulans tooth in occlusal view with simplified diagram to the right. The fixed landmarks are illustrated by large blue circles, sliding semi-landmarks by small red circles. The boundaries of the cusps and the stylids (small flat or saddle like surfaces joining cusps) are difficult to precisely identify, but have been illustrated in the diagram for clarity. (TIF) [file pone.0182565.s012.tif]

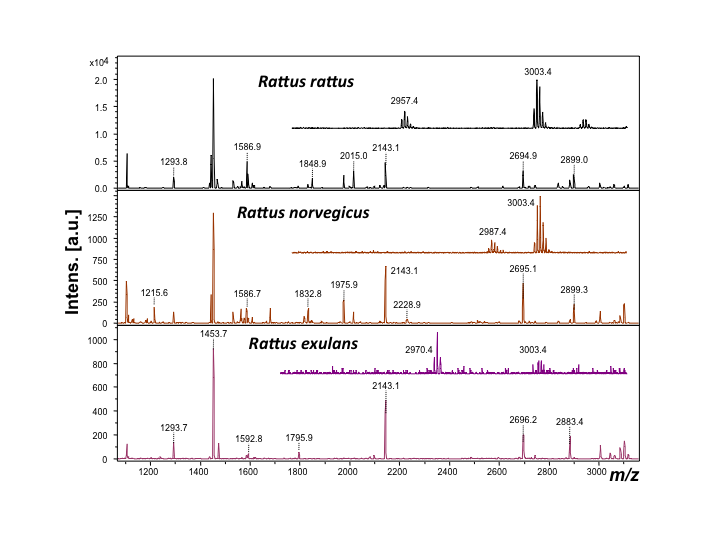

Supplement: S4 Fig — MALDI peptide mass fingerprint spectra of collagen tryptic digests from the reference bone material of Rattus rattus (top), Rattus norvegicus (middle) and Rattus exulans (bottom). (TIF) [file pone.0182565.s013.tif]

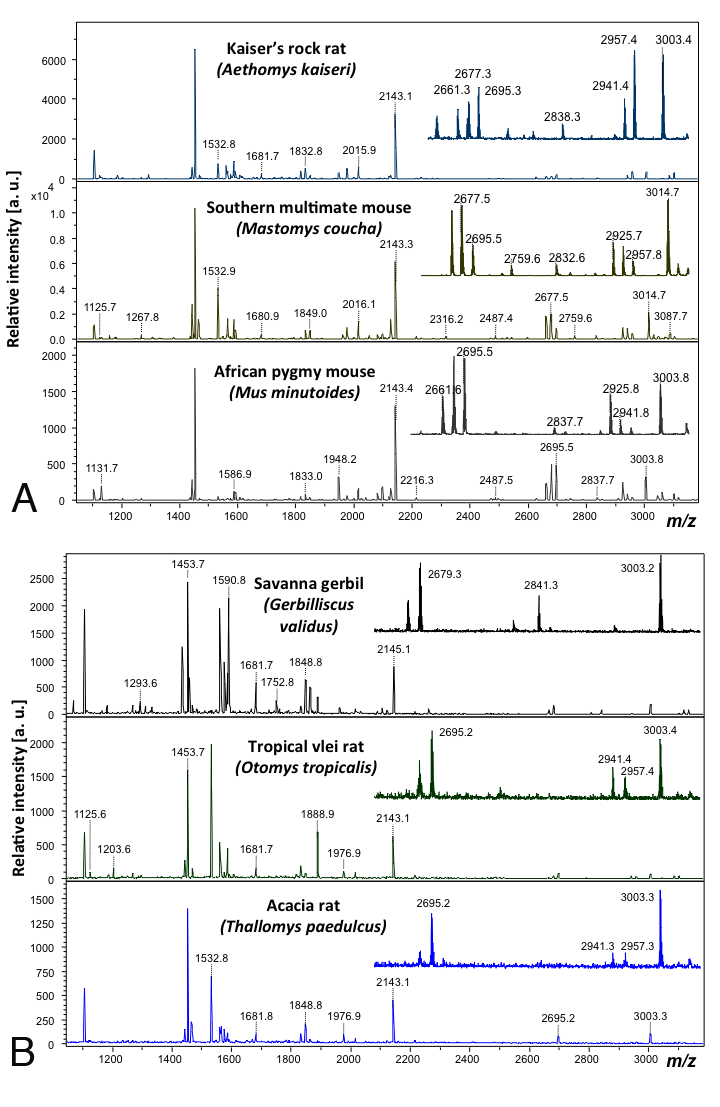

Supplement: S5 Fig — A: MALDI peptide mass fingerprint spectra of collagen tryptic digests from the reference bone material of Aethomys kaiseri (top), Mastomys coucha (middle) and Mus minutoides (bottom). B: MALDI peptide mass fingerprint spectra of collagen tryptic digests from the reference bone material of Gerbilliscus validus (top), Otomys tropicalis (middle) and Thallomys paedulcus (bottom). (TIF) [file pone.0182565.s014.tif]

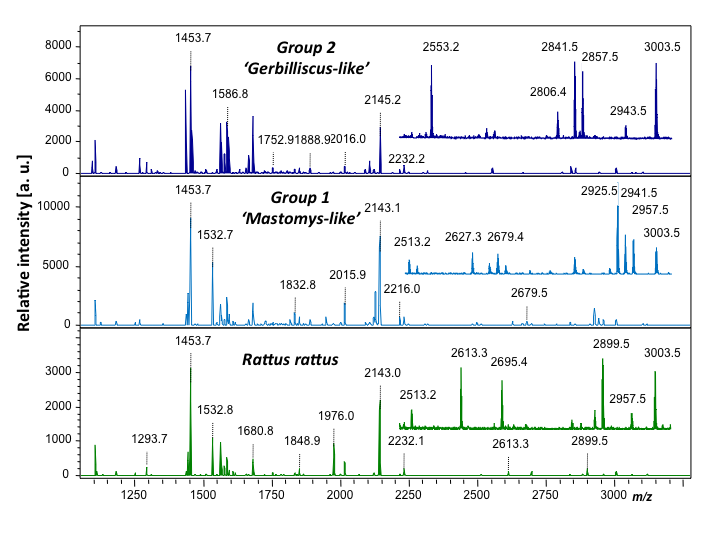

Supplement: S6 Fig — Example of MALDI peptide mass fingerprint spectra of collagen tryptic digests from the archaeological samples studied, showing the three most commonly identified types: Rattus rattus (bottom); Group 1 (middle), which most closely resembles Mastomys; and Group 2 (top), which most closely resembles Gerbilliscus. (TIF) [file pone.0182565.s015.tif]

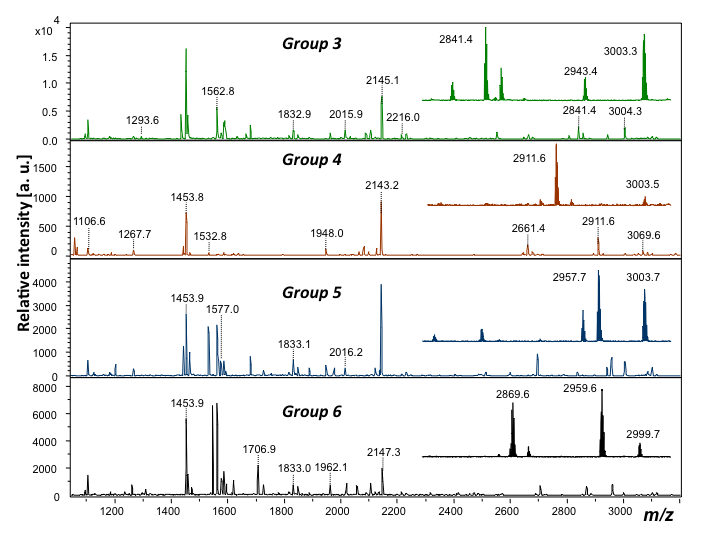

Supplement: S7 Fig — MALDI peptide mass fingerprint spectra of collagen tryptic digests from archaeological specimens that form groups of unknown taxa. (TIF) [file pone.0182565.s016.tif]
